# Longitudinal wastewater sampling in buildings reveals temporal dynamics of metabolites

**DOI:** 10.1371/journal.pcbi.1008001

**Published:** 2020-06-29

**Authors:** Ethan D. Evans, Chengzhen Dai, Siavash Isazadeh, Shinkyu Park, Carlo Ratti, Eric J. Alm

**Affiliations:** 1 Department of Biological Engineering, Massachusetts Institute of Technology, Cambridge, Massachusetts, United States of America; 2 Senseable City Laboratory, Massachusetts Institute of Technology, Cambridge, Massachusetts, United States of America; EMBL-Heidelberg, GERMANY

## Abstract

Direct sampling of building wastewater has the potential to enable “precision public health” observations and interventions. Temporal sampling offers additional dynamic information that can be used to increase the informational content of individual metabolic “features”, but few studies have focused on high-resolution sampling. Here, we sampled three spatially close buildings, revealing individual metabolomics features, retention time (rt) and mass-to-charge ratio (mz) pairs, that often possess similar stationary statistical properties, as expected from aggregate sampling. However, the temporal profiles of features—providing orthogonal information to physicochemical properties—illustrate that many possess different *feature temporal dynamics* (fTDs) across buildings, with large and unpredictable single day deviations from the mean. Internal to a building, numerous and seemingly unrelated features, with mz and rt differences up to hundreds of Daltons and seconds, display highly correlated fTDs, suggesting non-obvious feature relationships. Data-driven building classification achieves high sensitivity and specificity, and extracts building-identifying features found to possess unique dynamics. Analysis of fTDs from many short-duration samples allows for tailored community monitoring with applicability in public health studies.

## Author summary

Understanding a community’s wastewater profile may allow for specific and targeted interventions. Untargeted wastewater metabolomics provides a rich data source, but one that is high dimensional, noisy and difficult to understand. We analyze building-to-building differences and through-time patterns from temporal wastewater metabolomics data, obtained directly from three buildings. We develop and apply computational techniques to extract building-specific temporal and stationary properties for each small molecule feature. Stationary properties are predominantly conserved, but by studying the temporal dynamics, we find distinct, building-specific signatures and metabolite patterns. Interestingly, using clustering techniques and temporal similarity metrics, we find that for each building there exist groups of small molecules that possess highly similar temporal dynamics, despite having vastly different molecular properties (e.g. molecular weight or chromatographic retention time). These findings may suggest similar generative processes for such small molecules, which may lead to increased biological understanding. Additionally, our computational methods link putatively identified small molecules with unknown features. This produces a list of unknown compounds, with community-specific temporal dynamics for follow up experimental analysis and targeted discovery to better understand a community of interest.

## Introduction

Wastewater sampling presents a means to monitor the general health [1], chemical exposure [2,3], and size [4] of a population in a rapid and noninvasive manner. Many studies have been performed at wastewater treatment plants (WWTPs), as these sites are relatively easy to sample, and yield aggregate information on large populations, e.g. from an entire city [2]. As an example of the correlations that can be captured by such studies, an increase in antipsychotics, antidepressants and other therapeutic drugs was observed in wastewater between 2010 and 2014 in Athens, Greece during a time of significant economic turmoil in the country [5]. Given the proper sampling and analysis methods, wastewater can provide meaningful, community-specific public health information.

Most wastewater epidemiology and metabolomics studies have focused on the aggregate chemical load on large populations, typically using targeted metabolomics to acquire highly sensitive, context-specific information about select small molecules present at WWTPs. City- and country-wide studies have focused on monitoring licit [5,6] and illicit drugs [7–9], including sports doping agents [10,11], and have used these results to estimate public drug consumption [12]. Targeted applications have ranged from monitoring stress-related molecules [13], plasticizers [3,14], and pesticides [2] to metabolites associated with alcohol [15] and tobacco [16]; general population biomarkers [17]; and the environmental release of pharmaceuticals [18]. In addition to chemical identification, wastewater metabolomics can also be used to estimate population size [19,20]. However, aggregate analysis of large populations potentially misses public health-relevant information on temporal dynamics and sub-population characteristics.

There are multiple ways to incorporate temporal information in wastewater metabolomics that depend on sampling methods and location. One common route is to collect many composite samples, often over 24 hours, via extended continuous sampling and typically at sites of aggregated wastewater [4,21,22]. This route is logistically easier, as it only requires a single site when using WWTP-based sampling. However, the temporal component provides an averaged signal, even with multiple single day composite samples. An alternative, the approach we take here, is to perform close-to-source, short continuous sampling (or periodic grab sampling) without combination [23]. The second route often requires multiple locations and high sampling frequency (hourly to daily or near-daily), necessitating a large number of samples. Extensive sampling is required to alleviate the problem of signal noise and stochasticity associated with short sampling of small populations. However, a benefit of this approach is that it provides a precise temporal snapshot of the molecules present at a given time and, with longitudinal samples, temporal dynamics with minimal signal averaging. This sampling may provide information on individual contributions to community wastewater, and when compositional shifts or chemical exposure events occur.

We conducted a multi-month, untargeted metabolomics analysis of wastewater from three individual buildings to understand the information contained in longitudinal sampling of small populations. To study the temporal resolution needed to characterize a small population, we performed direct, intermittent sampling over three months from a multipurpose-use building (Building 1) and two residential buildings (Buildings 2 and 3). While through-time statistical values (mean intensities) of the features showed minimal differences between buildings, analysis of *feature temporal dynamics* (fTDs) uncovered extensive differences. Temporal feature clustering and modeling, internal to each building, revealed numerous groups of shared fTDs that often displayed random but large intensity fluctuations. These dynamics would likely be unobserved or averaged out using alternative sampling approaches. Similarity of fTDs suggested links from putative metabolites to unknowns as well as between features with drastically different mz and rt values, both within and across buildings. To extract additional building-distinguishing information, we trained interpretable machine learning (ML) models using daily feature profiles. Extending and generalizing our analysis methods, we found additional fTDs that correlated with those of select putative molecules, suggesting features for follow-up analysis.

## Results

### Traditional statistical approaches do not capture the full temporal differences between buildings

Feature summary statistics (mean and standard deviation through time of feature ion intensities) provide a simple method to conceptualize and coarsely categorize feature stability. This stratification allows one to triage the features according to the research question of interest. Using this approach, we identified stable and unstable features that were generally similar between buildings. Further subcategorization and analysis of the unstable features revealed unique day-to-day dynamics, suggesting that summary statistics do not fully capture temporal dynamics that are essential components of a small population’s wastewater metabolome.

#### Longitudinal multi-month sampling allows for temporal variation-based feature grouping

**Fig 1:**
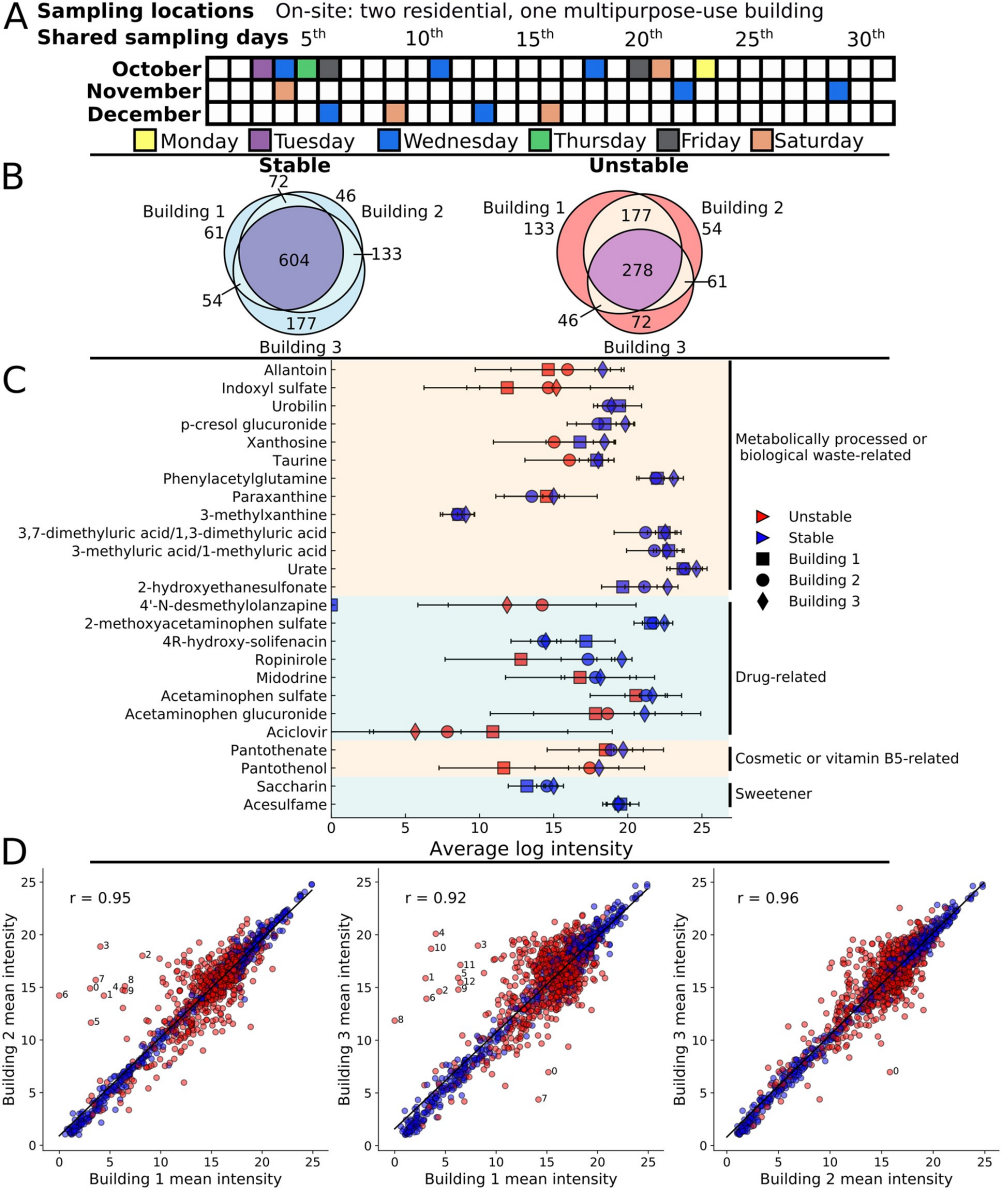
Multi-month sampling reveals numerous temporally stable and unstable features that generally possess comparable mean intensities across buildings. (A) Sampling timeline across three months for all buildings with shared sampling days filled with color. (B) Breakdown of the stable (through-time standard deviations < 2) and unstable metabolites and their overlap across the three buildings. (C) Average through-time mean log intensities and standard deviations of select features that were mapped to putative metabolites (level 2 identification); panel displays a selection of metabolites related to human activity, drugs, vitamin B5, and sweeteners. (D) Average through-time mean log intensity comparisons between all combinations of the three buildings. Red dots represent unstable features; blue dots represent features stable in at least one building. Building 1–2–0: N-decanoylglycine, 1: 237.9310Da/209.2619s, 2: girgensonine, 3: N-lauroylglycine, 4: 257.1955Da/491.0883s, 5: rhombifoline, 6: 4’-N-desmethylolanzapine, 7: 320.1906Da/475.7729, 8: 6-keto-decanoylcarnitine, 9: 445.0431Da/48.8418s; Building 1–3–0: 226.8621Da/33.2461s, 1: N-decanoylglycine, 2: 237.9310Da/209.2619s, 3: girgensonine, 4: N-lauroylglycine, 5: 257.1955Da/491.0883s, 6: rhombifoline, 7: acetylsulfamethoxazole, 8: 4’-N-desmethylolanzapine, 9: 1-[(3-chlorophenyl)methyl]-N,N-diethyl-3-piperidinecarboxamide^, 10: 320.1906Da/475.7729, 11: 6-keto-decanoylcarnitine, 12: 445.0431Da/48.8418s; Building 2–3–0: 226.8621Da/33.2461s. ^MS identity level 3, otherwise 2 for named compounds.

We observed two distinct groups of features during multi-month sampling of three spatially close buildings: temporally stable, and temporally unstable. Sampling occurred over three months, with the most dense sampling (multiple times per week) occurring in the first 3 weeks, followed by sampling approximately 1–2 times per week for the remainder of the period (Fig 1A). Liquid chromatography mass spectrometry (LCMS) produced 1425 features. 363 features were putatively identified at minimum reporting standard (MRS) [24] level 2 (primary mass matched to database and secondary mass spectrum matched to *in silico* fragmentation spectrum), 257 only at level 3 (primary mass matched to database), while most (805) were unannotated (level 4). The features were separated by through-time standard deviation; analyzing the distribution of standard deviations, two peaks were observed (one at ~1 and the other > 2, S1 Fig) for which stability cutoff values were set to separate these cases; values < 2 were considered stable, and ≥ 2, unstable. For both categories, the majority of features could not be annotated with a database chemical class, subclass or direct parent; apart from this, many different and generally low metabolite count subclasses were observed, with the most prevalent being: amino acids, peptides and analogues, carbohydrates (and conjugates) as well as fatty acids and conjugates (Methods, S2 Fig). Of the stable features, 53% were stable in all three buildings (Fig 1B). While only 34% of the unstable features were unstable in all buildings, with large fractions uniquely unstable in one or two buildings (Fig 1B).

Untargeted metabolomics revealed numerous putative human and human-associated small molecules, both in the stable and unstable categories, that displayed a range of through-time mean intensities, standard deviations, and building-to-building variability. We observed several chemicals and common metabolic products of human activity, including metabolites from caffeine metabolism (xanthine-based metabolites [25]), dietary tryptophan processing (indoxyl sulfate [26]) as well as expected urinary metabolites (urate [27], phenylacetylglutamine [28], and 2-hydroxyethanesulfonate [29]—Fig 1C). The majority of these metabolites were stable in all three buildings, with the exception of indoxyl sulfate, which was unstable across all three buildings. Putative drug-related metabolites displayed substantial feature instability and primarily consisted of acetaminophen metabolites plus possible drugs for restless leg syndrome (ropinirole) and low blood pressure (midodrine). Putative artificial sweeteners (acesulfame and saccharin) appeared stable across the three buildings, but chemicals naturally found in humans as well as in many health and cosmetic products (pantothenate (vitamin B5) and its precursor pantothenol—Fig 1C) displayed high variability. A large number of the drug- and cosmetic-related features appeared unstable in many buildings, particularly in Building 1.

Through-time statistical analysis suggested that individual features often appeared at similar mean intensities for all three building-to-building comparisons—especially the stable features. Linear relationships were observed for feature intensity comparisons between all buildings (R = 0.95, 0.92 and 0.96 for the Building 1-to-2, 1-to-3 and 2-to-3 comparisons, respectively—Fig 1D). While these high correlation coefficients were calculated using all features, the unstable features were more dispersed, and minimally correlated between buildings. Several unstable features appeared at low intensities in Building 1 relative to the other two, including 4’-N-desmethylolanzapine (a benzodiazepine) and two acyl-glycines among others (Fig 1D). Outside a lack of specific prescription drug use, it is challenging to explain such differences in intensities. Aside from these few cases and assuming consistent ionization across samples, these data suggest that many features exist at comparable average concentrations in buildings with different populations.

Statistical analysis between weekends and weekdays only uncovered statistically significant features when all weekdays were included. Comparing features from only Saturdays to Wednesdays using false discovery rate (FDR) corrected Mann-Whitney U-test (MW) P-values (Q-value), no features with Q < 0.05 were observed in any buildings (S3 Fig). However, including the remaining weekdays, Building 1 displayed hundreds of significant features while the other two buildings possessed no significant features. For these significant features, the largest subclasses included: amino acids, peptides and analogues, fatty acids and conjugates along with carbohydrates and their conjugates. However, the majority of features were not annotated or possessed no chemical class annotation.

#### Few temporally stable features are statistically different between buildings using a stringent cutoff

**Fig 2:**
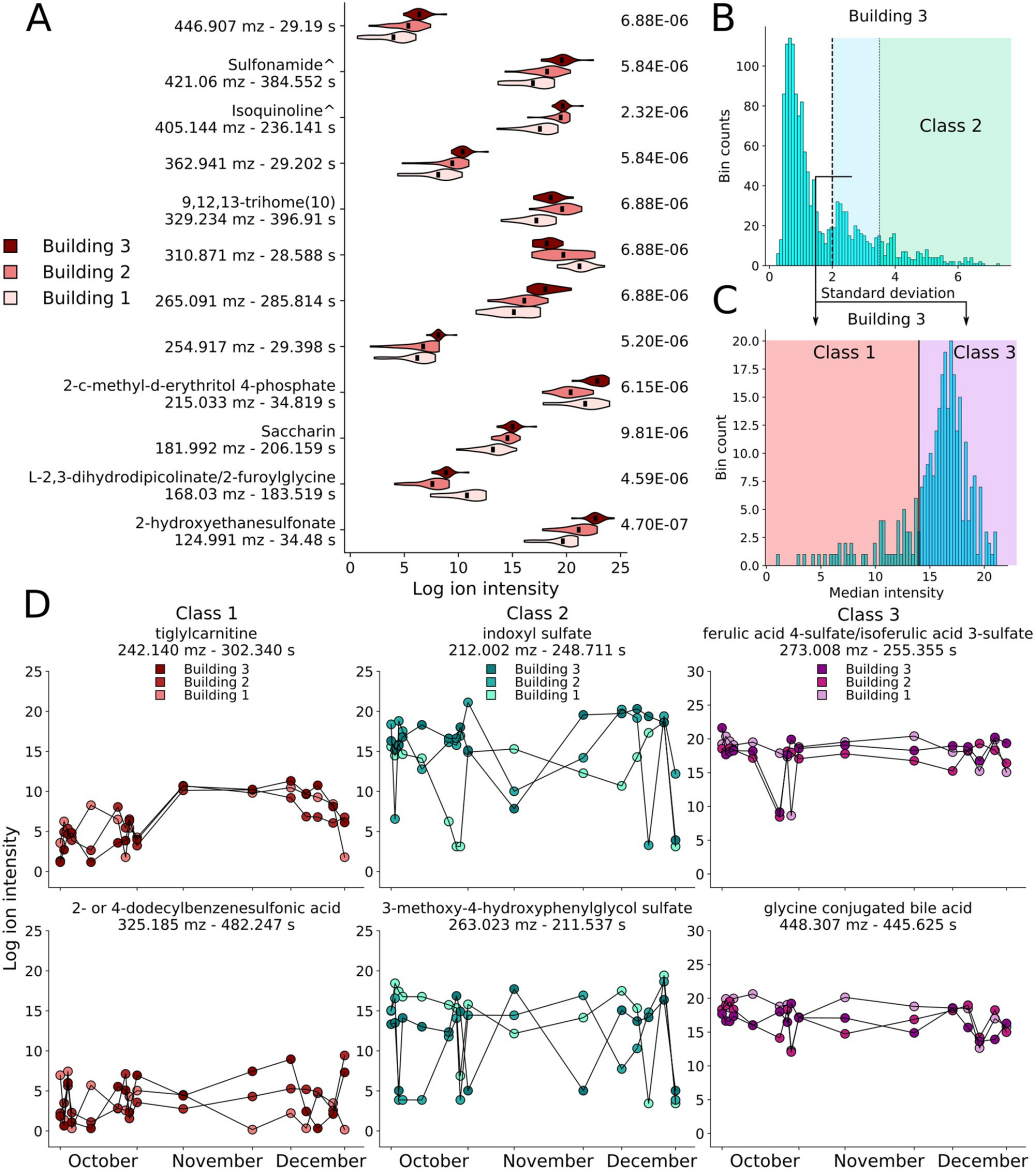
12 stable features are statistically different between buildings, and unstable features display three temporal classes. (A) Violin plot of all 12 significant features depicting building-to-building differences, distributions and FDR corrected P-values (right column). Names are levels 2 or 3 putative identifications (^ depicts level 3; Sulfonamide: N’-(3-chloro-2,4,6-trifluorophenyl)sulfonyl-1-adamantanecarbohydrazide, Isoquinoline: 2-[[2-[2-(2,3-dihydroindol-1-yl)-2-oxoethyl]-1-oxo-5-isoquinolinyl]oxy]acetic acid ethyl ester, S2 Table). (B) Unstable feature separation for Building 3, depicting features with through-time standard deviation in excess of 3.5 (Class 2) and those below. (C) Unstable feature separation for Building 3, showing features with median log intensity separated by a median intensity of 14 (Class 1). (D) Unstable feature time courses for select metabolites of the three classes: Class 1 features (left column); Class 2 features (middle column); and Class 3 features (right column, S3 Table).

Only 12 stable features were present at significantly different mean levels between the three buildings (FDR corrected Kruskal-Wallis, KW, P-value < 0.00001). This corrected P-value was chosen to analyze only the ‘most significant’ features between buildings; however, a range of values were explored (for all features, stable features, and unstable features, S4 Fig). This cutoff may be altered for studying different communities. Of the 12 significant features, 9 were observed at higher levels in Buildings 2 and 3 relative to Building 1 (Fig 2A). Unlike manually searching for specific metabolite types, statistical analysis found the urine metabolite 2-hydroxyethanesulfonate and sweetener saccharin among the significant features. Chemical classification proposed that the two level 3 identified features corresponded to a isoquinoline and a sulfonamide. Few commonalities were observed between these features as they appeared across a wide range of mz, rt, and intensity values.

#### Unstable features can be further split into three dynamics-based classes

Temporal analysis of the unstable features suggested three general temporal profiles, providing a more fine-grained classification, and a means for conceptualizing their dynamics (Fig 2B–2D, S3 Table). Class 2 unstable metabolites possessed through-time intensity standard deviation levels greater than 3.5; this cutoff was used to separate the unstable features in the second peak of the standard deviation distribution from those with even higher variability (Fig 2B and S1 Fig). Representative examples of Class 2 include indoxyl sulfate and the sulfate-modified neurotransmitter-derived metabolite 3-methoxy-4-hydroxyphenylglycol (Fig 2D). Class 1 contained features with a through-time median intensity less than 14 (after removal of the Class 2 features), this value separated the main group of unstable features from those with low overall intensities (Fig 2C and S1 Fig). This class included metabolites that are typically observed at low levels in wastewater, but that are occasionally present at high levels. Class 3 consisted of the remaining features possessing mid-level log intensities with irregular intensity changes. Class 3 included both a ferulic acid sulfate and putative glycine conjugated cholic acids (Fig 2D). Chemical classification of these three temporal profiles found the majority to be unannotated with the remainder, generally, binned into classes, subclasses and direct parent groups with single membership, suggesting no increased prevalence of any one chemical class (S5 Fig). This scheme of subclassification may be transferred to alternative data, for which the specific cutoff values should be re-tuned. Temporal analysis departs from using a single statistical parameter to describe a time-series; using only three simple groups highlighted different feature temporal dynamics (fTDs)—this prompted a more comprehensive dynamics analysis.

### Characterizing and modeling the dynamics of individual buildings with clustered fTDs

Temporal analysis supplied a more nuanced view of the features, and thus buildings, than general stability types. To characterize the extent of temporal dynamics, we used unsupervised clustering which revealed a large diversity of feature dynamics in a community’s wastewater metabolome. To understand whether one sampling period could be predictive of another for the same building, we fit clusters with a Gaussian process (GP), the results of which suggested that dynamics, particularly large deviations from the mean, cannot be predicted on days not sampled. Such modeling highlighted the importance of frequent temporal sampling. Differences between buildings were further highlighted by focusing on the temporal profiles of putative classes of molecules related to metabolism and lifestyle.

#### Clustering fTDs uncovers groups of features with highly similar temporal dynamics

**Fig 3:**
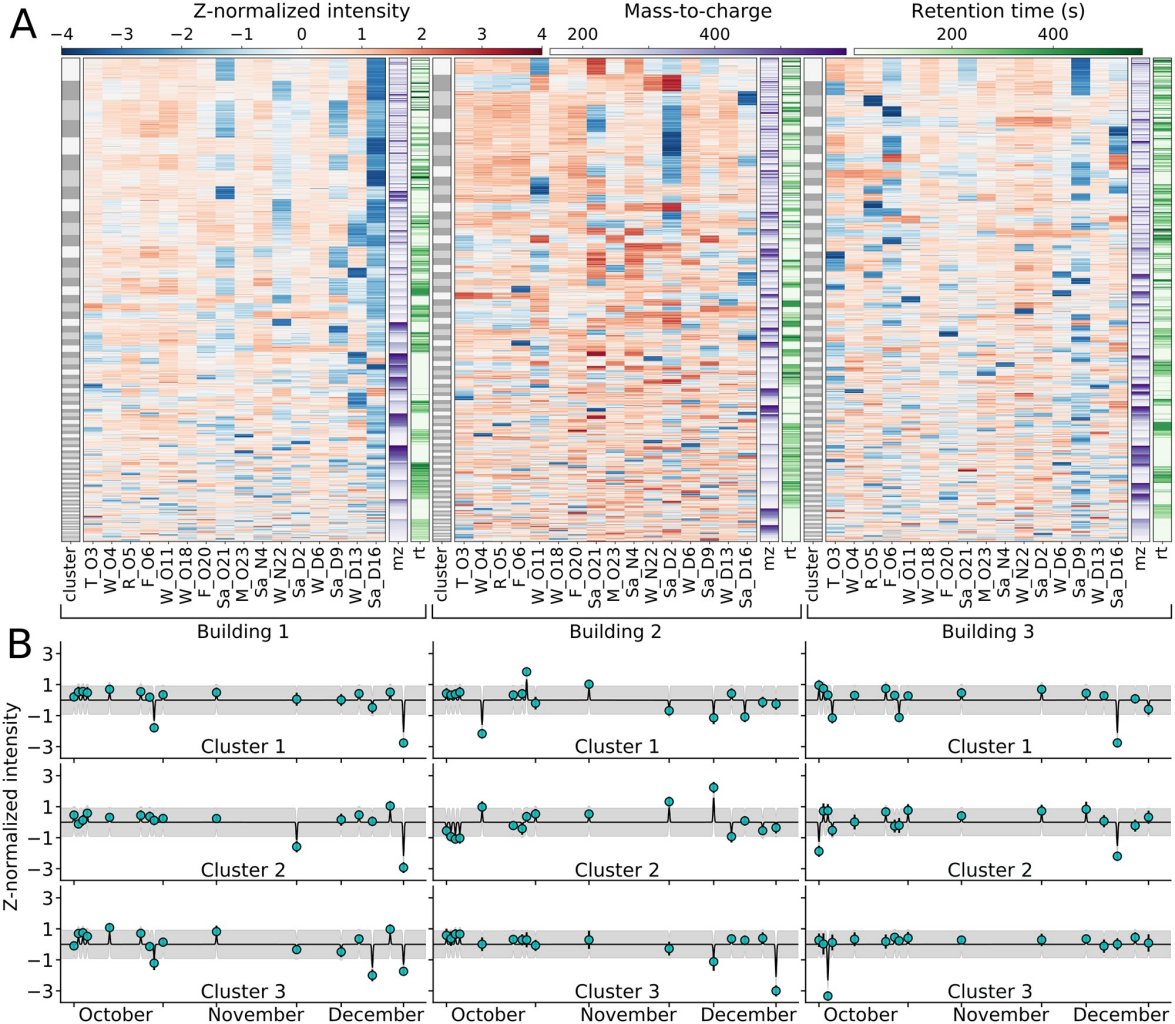
Individual buildings possess unique fTDs with most dynamics governed by a few clusters and large, single day deviations from the mean. (A) Summary of all building data sets using only shared sampling days. Gray columns show the 100 clusters, with individual box heights corresponding to cluster size. The 16 red and blue columns depict single-day, z-normalized log intensities for each feature (individual rows) for the three buildings; purple and green columns show feature mz and rt respectively. Color keys shown above the plots. (B) Average cluster values (teal points) for the three largest clusters for each building, with each cluster fit to a GP. Model standard deviation in gray.

K-means clustering of individual building features displayed several prominent families that differed between buildings. One hundred clusters were used to group z-normalized features, providing highly similar intracluster dynamics, mostly within the range of -1 to 1 (Fig 3A). The high temporal sampling revealed that many clusters exhibited sudden, single-day spikes or drops in intensity (Fig 3A, dark blue and red regions). Additionally, many days displayed similar intensity patterns across-clusters; for example in Building 1, on October 6^th^ the top 8 clusters showed similar z-normalized intensities (not a comparison of the absolute intensities), and on Saturday, December 16^th^ the majority of clusters demonstrated a general drop in normalized intensity for many features. When comparing across the three buildings, no obvious trends were observed in terms of mean cluster dynamics, which varied between buildings. The majority of clusters in all buildings were composed of features spanning a large range of mz and rt values (Fig 3A purple and green columns). Several of the largest clusters were primarily made from features lacking annotation (Building 1: clusters 2 and 5; Building 2: clusters 1 and 2; Building 3: clusters 1 and 3, S6–S8 Figs). The remaining top clusters were composed of different chemical classes and subclasses, the majority of which possessed one or two counts with no clear (sub)class preferences.

GP cluster modeling displayed minimal day-to-day predictive power, and mean reversion, suggesting that high frequency (daily) sampling is important for capturing the observed unpredictable alterations. As a previous 24-hour metabolomics analysis [23] demonstrated strong diurnal patterns in wastewater, we used a small length scale parameter for the GP. Using a conservative 3-hour length scale, information decayed within hours following perturbation, providing no day-to-day predictive power (Fig 3B). Modeling the observed data mixed with additional theoretical buildings (i.e., sampling further downstream or with longer sampling periods, S1 Text for methods), demonstrated that for feature clusters, the probability of observing large deviations from the mean drops with only tens of additional buildings (S9 Fig). These results highlight the importance of short, high-frequency, and close-to-source sampling; this indicated that our own sampling scheme likely missed large wastewater dynamics.

#### Frequent sampling allows for tracking dynamics of human-related metabolite groups

**Fig 4:**
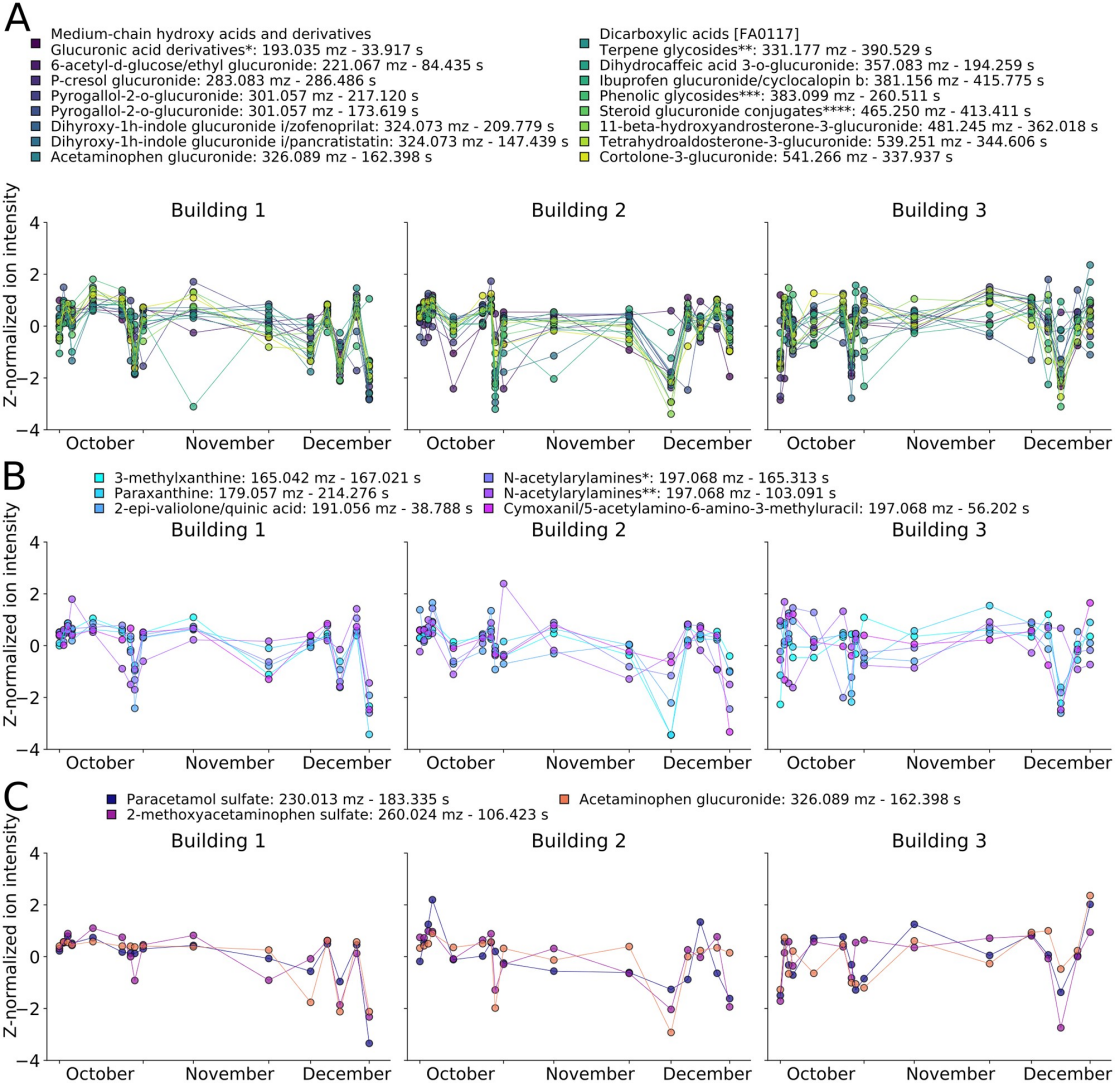
High temporal sampling allows for monitoring putative metabolite levels and dynamics in a building-specific manner. Z-normalized time courses of metabolites with MS level 2 identity, subclass names are supplied when several putative metabolites matched for (A) glucuronide-related, *5-dehydro-L-gluconate / D-glucuronic acid / iduronic acid / 5-keto-D-gluconate, **neomenthol-glucuronide / (2s,3s)-2-hydroxytridecane-1,2,3-tricarboxylate / lmfa13010036 / ***5-(3’,5’-dihydroxyphenyl)-gamma-valerolactone 3-o-glucuronide / 5-(3’,4’-dihydroxyphenyl)-gamma-valerolactone-4’-o-glucuronide, ****3-alpha-hydroxy-5-alpha-androstane-17-one 3-d-glucuronide / etiocholanolone glucuronide (B) caffeine-related, *5-acetylamino-6-amino-3-methyluracil / 6-amino-5[n-methylformylamino]-1-methyluracil, **5-acetylamino-6-amino-3-methyluracil / 6-amino-5[n-methylformylamino]-1-methyluracil and (C) acetaminophen-related putative metabolites across the three buildings. Full metabolite details can be found in S4 Table.

The three buildings displayed different dynamics for specific human and lifestyle-related putative metabolite groups. The groups studied included glucuronide-modified compounds [30], caffeine-related metabolites [25], biologically modified acetaminophen [31], along with glucoside conjugated molecules (Fig 4, S10 Fig, and S4 Table for full names). These human-associated putative groups possessed diverse temporal dynamics in each group and building. For instance, many of the features showed large changes across buildings; however, the days on which the specific dynamics occurred differed for each given putative metabolite. Within each building, select putative metabolites from each group displayed similar temporal dynamics, perhaps due to similar biochemical processing (e.g. different metabolites of acetaminophen). However, not all temporal profiles in a group were always similar, for instance the October levels of many glucuronides and caffeine metabolites displayed pronounced differences. While the ability to identify additional features is required for larger, targeted chemical tracking, this analysis highlighted the potential of high-frequency wastewater sampling to monitoring groups of health and lifestyle-related compounds.

### Temporal data draws new feature correlations

Analyzing individual fTDs within and between buildings allows comparison of building-to-building similarity or lack thereof. To do so requires calculating feature similarities using through-time distance measurements or correlations. These methods reveal features and clusters that are correlated and others that are anticorrelated, something not necessarily possible with single time point measurements.

#### Different buildings show few similar fTDs while many are correlated within a building

**Fig 5:**
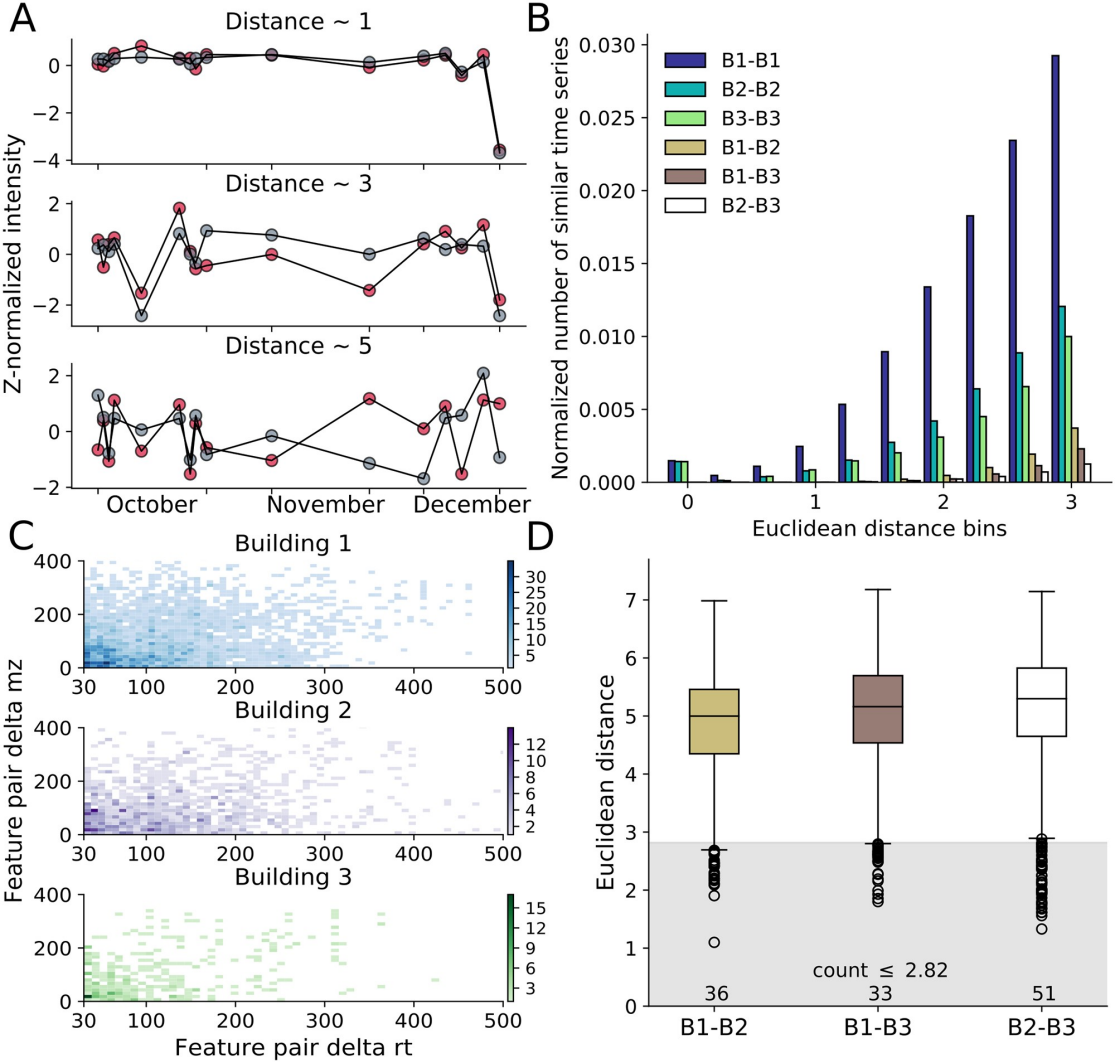
Many features display highly similar dynamics within, but not between buildings, with a large number being unrelated in mass and retention time. (A) Example distance plots for z-normalized data for distances of ~1, 3 and 5. (B) Histogram of all-to-all feature Euclidean distance calculations for different building pairs. (C) Within-building two dimensional histograms of all-to-all feature comparisons for which the Euclidean distance was < 1.5 and the difference in retention time was > 30 s, plotted versus the feature pair’s delta mz (y-axis). (D) Box plot of Euclidean distances between identical features across different buildings. The gray region holds all features below a distance of 2.82.

A large number of highly similar fTDs were observed internal to buildings but almost no similarities were observed between buildings. To study the similarity between z-normalized time series, we analysed feature pair time series at different Euclidean distance cutoffs (example distances shown in Fig 5A). We chose two similarity cutoff thresholds; 1.5 for high stringency and 2.82 for lower stringency similarity. These values can be tuned; here Building 1 primarily set the two thresholds, as a distance of 2.82 included a large 9.6% of pairs, while the 1.5 cutoff included 1.1% (S11 Fig). Buildings 2 and 3 both showed lower percentages for these cutoffs, with each generally possessing fewer similar time series and an increased mean distance for all pairs. A histogram of distances between all time series (an all-to-all comparison), displayed a greater fraction of similar time series within each building than between buildings, for which there were few similar time series even up to a Euclidean distance of 2.82 (0.70%, 0.36% and 0.25% for B1-B2, B1-B3 and B2-B3 comparisons respectively, Fig 5B, S12 Fig). Building 1 showed more intrabuilding similar time series than buildings 2 or 3, likely due to the decrease in intensity of hundreds of features on the final day, which would dampen the z-normalized intensities for other days (Fig 2D). The high intrabuilding zero-distance bin primarily corresponded to distances calculated between a feature and itself, plus a small number of very similar features. For the different comparisons, the majority of similar time series belonged to pairs in which both features had high average intensities; few belonged to pairs in which one feature had low average intensity, possibly due to instrumental noise (S13 and S14 Figs). This observation suggested that high similarity between fTDs did not arise from comparisons to normalized background or noise features.

Further analysis on the large number of similar fTDs within a building, revealed that many of the feature pairs (at the high stringency cutoff) possessed large differences in mz and rt (Fig 5C, S15 Fig for complete range). A 2-D histogram of only those feature pairs that differed by at least 30 s in rt showed that many of the shared temporal dynamics differed by 30–100 s and 0–100 Da. A large number of feature pairs corresponded to mz and rt differences much greater than 100 s and 100 Da; overall, feature similarities were observed across the full mz and rt domains (S15A Fig). A comparable, but more populated, 2-D histogram was obtained with the low stringency similarity cutoff (S15B and S15C Fig).

The distance between a feature and its corresponding feature across buildings (a one-to-one comparison) demonstrated that only a few features (36, 33 or 51) possessed similar temporal dynamics between buildings, even at the more inclusive, low stringency cutoff (Fig 5D). This analysis revealed that the majority of features displayed markedly different temporal dynamics, as the median distance was larger than five for each comparison.

**Fig 6:**
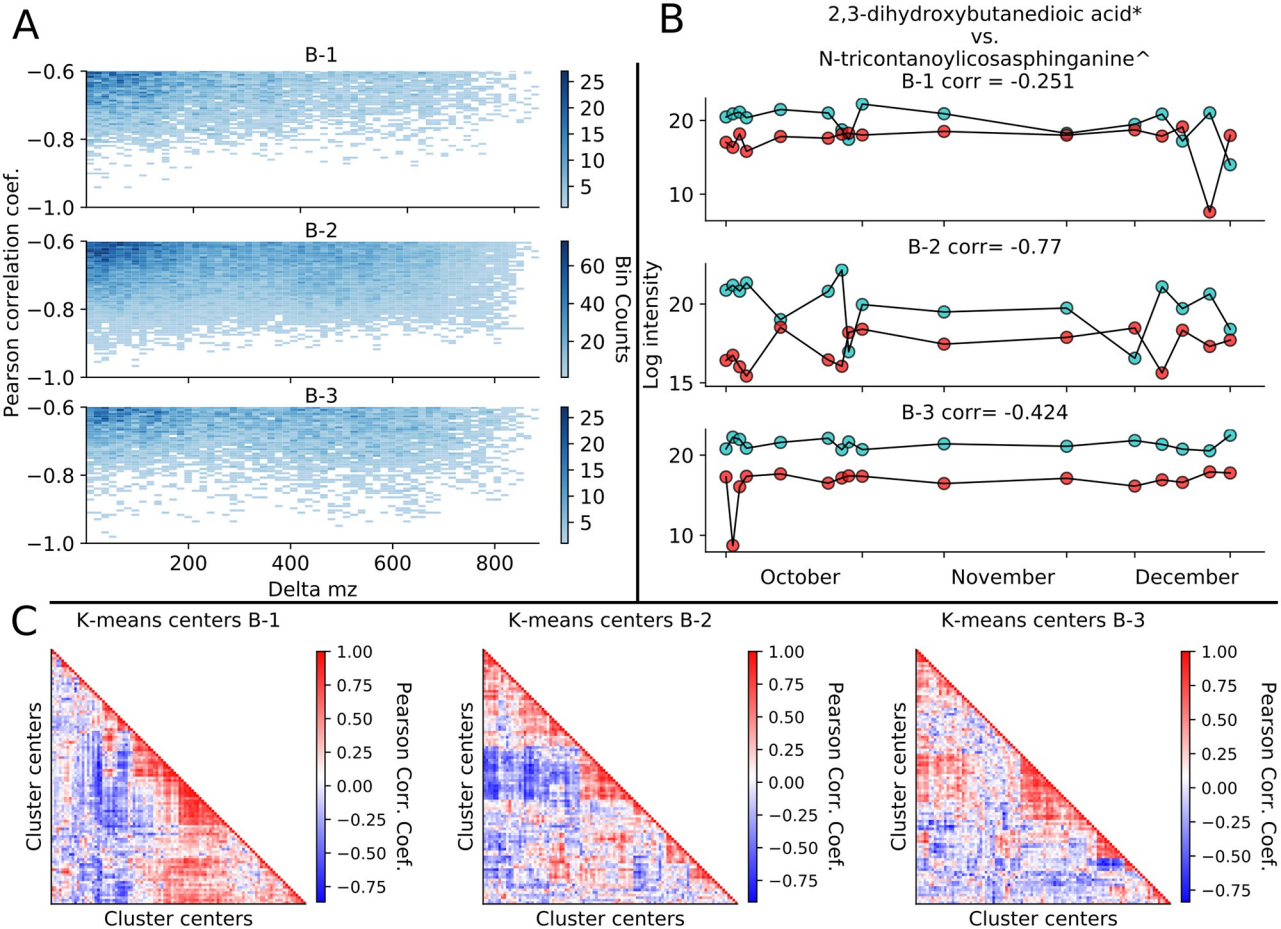
Individual building features and K-means cluster centers show anticorrelated temporal patterns. (A) 2-D histogram of pearson correlation between features versus their mass difference for each building showing only the anticorrelated region. (B) Example temporal profile of two features showing anticorrelated temporal behavior. Blue trace—2,3-dihydroxybutanedioic acid, red trace—N-tricontanoylicosasphinganine; * alternative compound—(s,s)-tartaric acid, ^ identification level 3. (C) Hierarchically clustered K-means centers with pairwise Pearson correlation coefficient.

Time series analysis revealed features and K-means cluster centers that are anticorrelated in time. Thousands of z-normalized feature pair time series (6750, 32453, and 7851 for Buildings 1, 2, and 3 respectively), some with large mass-to-charge ratio differences, possessed Pearson correlation coefficients less than -0.6 (Fig 6A, see S16 Fig for the full -1 to 1 range). 19505 of the 1014600 possible (non-identical) feature pairs had negative (< -0.2) correlation coefficients in all three buildings, for which Fig 6B shows one example. A smaller number of feature pairs (657, 4642, and 1187 from Buildings 1, 2, and 3 respectively) displayed negative Pearson correlations (< -0.6) but with time series statistical properties that were not significantly different (Q-value > 0.05), these anticorrelated pairs would not have been found with aggregate, single time point sampling (S17 Fig). Between pairs of buildings, 12 features showed anticorrelated behavior with a coefficient < -0.6; the putatively identified reduced riboflavin was anticorrelated in buildings 1 and 3 as well as 2 and 3 (S18 Fig). In addition to individual features, feature cluster centers from the K-means clustering showed both positive and negative (< -0.6) correlation coefficients (Fig 6C). While a number of such correlations may be due to small fluctuations of stable features, this would suggest anticorrelated temporal profiles exist, representing a benefit of temporal measurements.

### Machine learning classifies buildings and finds building-specific feature dynamics

Orthogonal to the temporal clustering approach, the complete feature profile of a single day provides a means for isolating community-specific feature information. Using an alternative objective—across-day building classification—with simple machine learning models, it is possible to extract features with unique building-to-building temporal patterns.

#### Single day feature profiles allow for building classification and find building-specific fTDs

**Fig 7:**
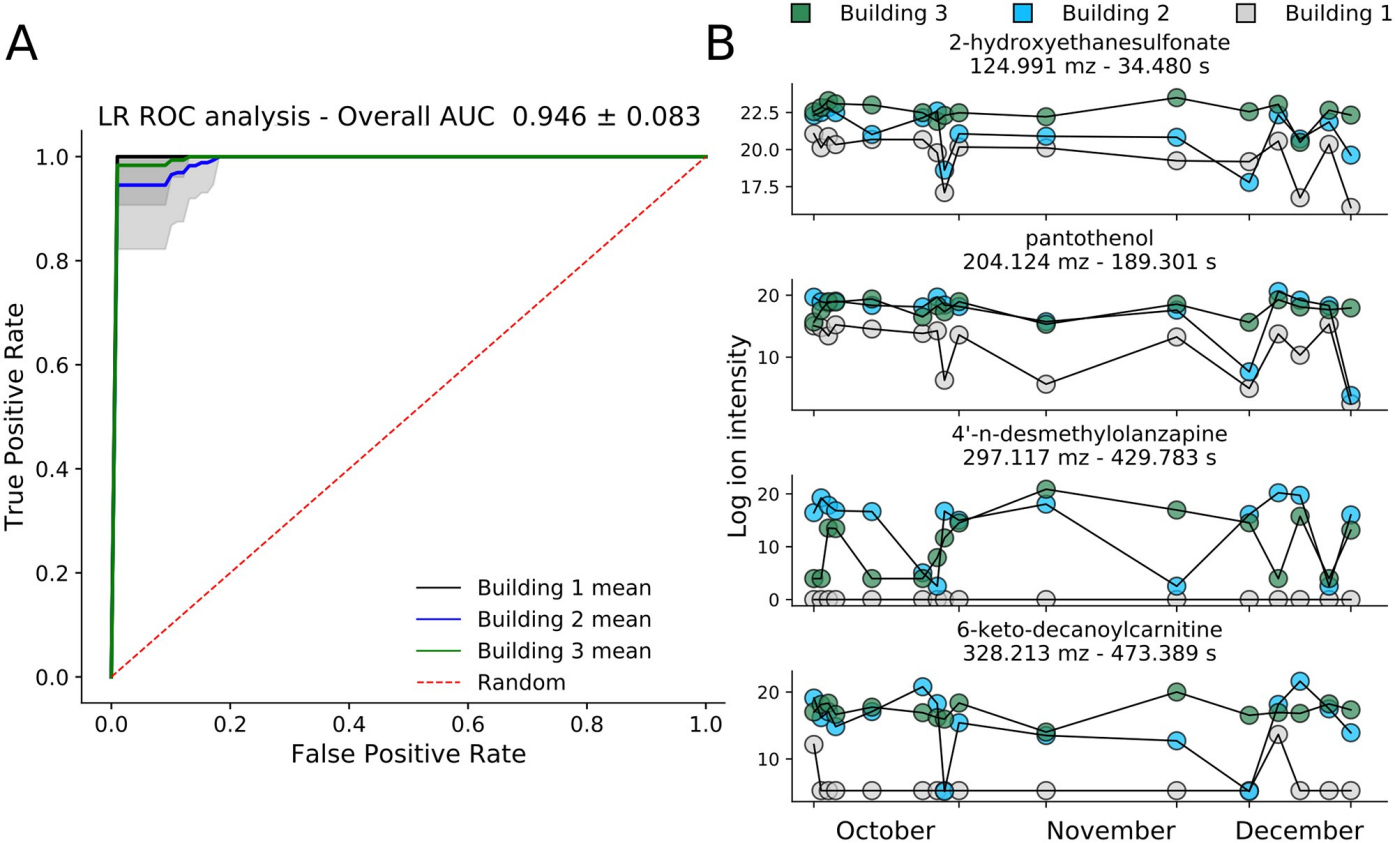
Single-day feature profiles allow accurate building classification and uncover features with building-specific fTDs. (A) ROC-AUC plot (true positive versus false positive rate) for the three one-versus-the-rest building comparisons, using individual days as data points with associated ion intensities as features. AUC plots and scores come from 50x repeated model training on randomized train-test data splits. (B) Putative identification (level 2) and log ion intensity time courses of four select, high-importance metabolites that contribute to model performance.

L1-regularized logistic regression (L1-LR) and random forest (RF) models provided high classification performance and revealed building-differentiating features. We used a one-versus-the-rest approach for model training, for which each input corresponded to all feature values from a single day. Importantly, we used standardized log-transformed features intensities calculated across all three buildings combined, not individual temporal z-normalized values. Receiver operating characteristic (ROC) area under the curve (AUC) analysis demonstrated high building classification AUC for all three comparisons with L1-LR models (0.946 ± 0.083 mean and standard deviation of 50-fold repeated model training, Fig 7A). The RF model did not perform as well (AUC = 0.906 ± 0.065, S19 Fig), but provided additional insight from the set of features used. Additionally, in line with statistical analysis, we found it possible to differentiate weekdays from weekends in Building 1 using an L1-LR model (AUC = 0.760 ± 0.180) but not in Buildings 2 and 3 (AUC = 0.484 ± 0.179 and 0.410 ± 0.233 respectively).

Features that were used in at least 40 of the independent L1-LR models and that possessed an average importance value greater than 0.005 across all 50 RF models demonstrated unique building dynamics. Four such feature time series are depicted in Fig 7B, of all which have been highlighted by alternative methods. The urine-related metabolite 2-hydroxyethanesulfonate and pantothenol both displayed lower ion intensity levels in the multipurpose-use Building 1 than in the residential buildings; similarly 6-keto-decanoylcarnitine, a urine metabolite used in non-muscle invasive bladder cancer diagnostic models [32], was mostly absent in Building 1 but appeared at high levels in the other two (Fig 7B). Such differences, for many features, in Building 1 relative to 2 and 3 may explain the ease of Building 1 classification. Beyond these four, many of the important metabolites were either stable and statistically significant between buildings, or possessed alternative unstable metabolite class labels (S5 Table). While carboxylic acids (their derivatives) and fatty acyls were the largest named classes, along with amino acids, peptides and analogues being the main subclass, the most prevalent group was that lacking any classification (S20 Fig). This minimally biased modeling, largely recapitulated the findings of traditional statistical and temporal analyses, and suggested metabolites that through subsequent temporal analysis were shown to possess unique and building-differentiating dynamics.

### Grouping temporally similar features suggests targets for follow-up studies

We extracted additional features that were temporally related to the set of metabolites identified by our analyses. We grouped additional features temporally similar to each of the select metabolites for all three buildings, and analysed between-building, feature-pair cluster co-occurrences. This directly suggested features, and possibly hypotheses, for specific follow-up experiments, and may comment on shared controlling processes (chemical, biological, etc.) that govern feature dynamics.

#### Intrabuilding temporal similarity and interbuilding co-clustering link possibly unrelated features

**Fig 8:**
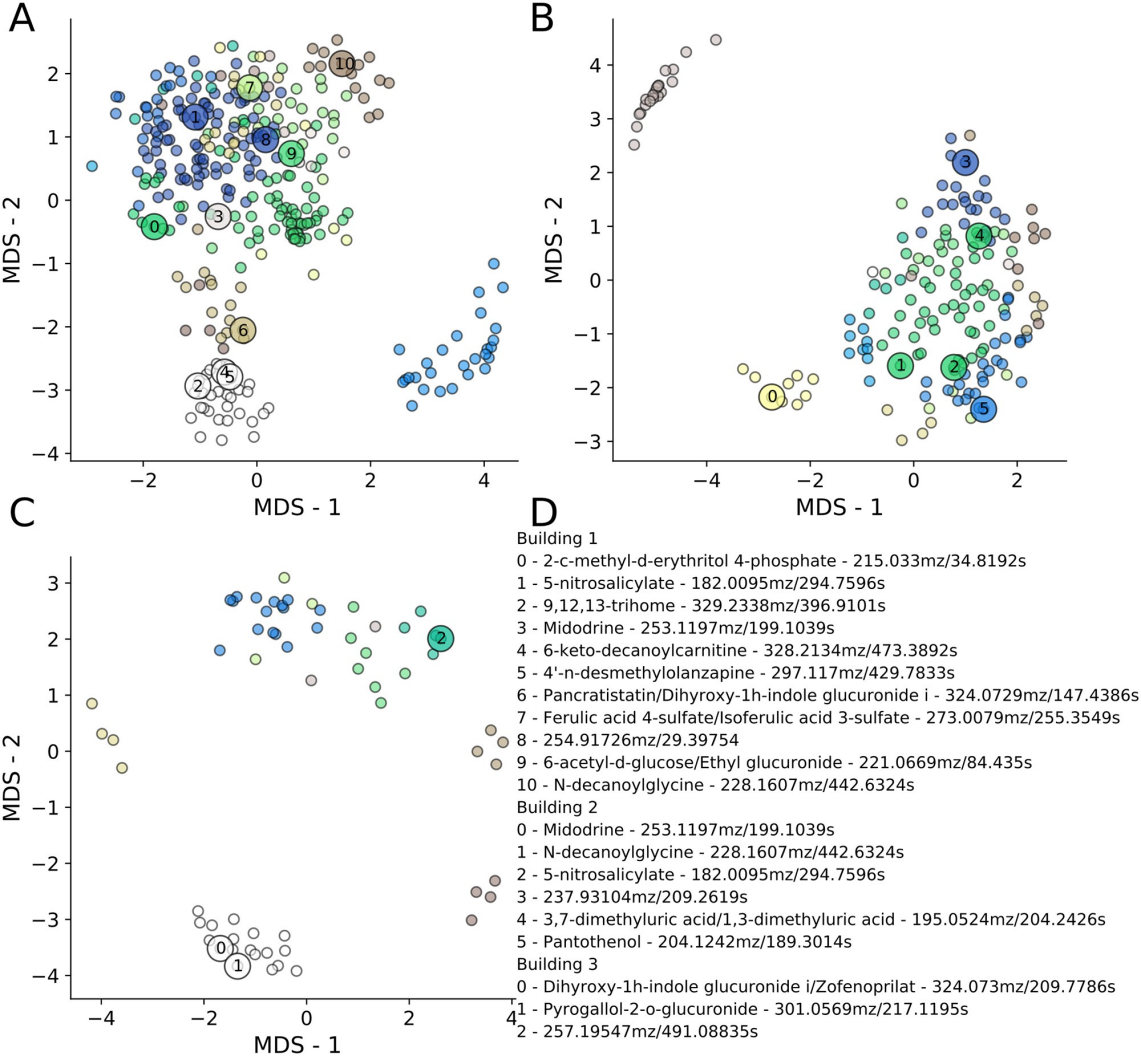
MDS analysis finds many putative metabolites or features that are temporally similar to select important features. MDS analysis of (A) Building 1, (B) Building 2, and (C) Building 3 feature groups with > 15, 8 or 3 (A–C respectively) similar features (as defined by intrabuilding feature-to-feature distance calculations). The ‘important features’ (IFs), to which distances from all other features were calculated, originated from the machine learning models, stability types, and other putatively named metabolites. Colors correspond to features that possess a Euclidean distance < 1.5 and group together. IFs are shown with numbers and large circles, and are labeled in (D). All names are level 2 identifications, unless ending with an ^ corresponding to level 3 identification with ppm error.

Analyzing the ‘important features’ (IFs, S2–S8 Tables) highlighted by our methods, we found that there were numerous, likely unrelated features within buildings that were highly correlated to the IFs. The IFs included the ML model features, putatively named metabolite groups, select unstable features, statistically significant stable features, and the urine, drug, sweetener, and cosmetics-related features. Metric multidimensional scaling (MDS) of the groups of features sharing fTDs with the IFs showed varying levels of clustering and co-clustering (Fig 8). Because many features were shared among multiple clusters, but only assigned to the largest (see Methods), many groups displayed overlap in this two-dimensional space. Similar to other methods, MDS revealed that many features and putative metabolites with large differences in mz and rt values grouped with some of the most prominent IFs, many of which are believed to be human-related (Fig 8D). The clusters suggested unknown features that may originate from the same source, for which additional analysis is needed for identification.

Between buildings we observed many co-clustered sets of features, despite most co-clustered features possessing different dynamics in each building. Using the intrabuilding K-means clusters, we found intersecting sets of co-clustered features between pairs of buildings (S21 Fig). Again, this revealed groups of features with substantially different mz and rt values and that co-clustered in either two or all three buildings. The co-clustering of specific feature groups across buildings, despite building-specific temporal dynamics (Fig 5D), lends additional credence to the observed associations. Co-clustering across buildings, along with MDS analysis, highlighted numerous, seemingly unrelated features that displayed similar temporal dynamics, suggesting specific compounds or unidentified mz and rt pairs for follow-up analysis. Thus fTD analysis may further our understanding of specific chemicals (e.g. pesticides or drugs) by suggesting other small molecules that would otherwise appear unrelated, but that may have been introduced into the waste stream by similar controlling processes.

## Discussion

Previous temporal studies of wastewater metabolomics have examined seasonal variation [33] or larger, aggregated populations at multiple time scales (often 24-hour aggregates), using downstream wastewater treatment plant (WWTP) sampling [16,34–36]. Here, we demonstrated the utility of short duration, high frequency (daily) direct building sampling to understand through-time statistical properties, individual feature temporal dynamics (fTDs), and clusters of temporally related features of single-building wastewater. Adding this temporal component, without substantial wastewater aggregation, may benefit future wastewater metabolomics studies by revealing community-specific metabolite dynamics and the daily burden of environmental pollutants, drugs, or lifestyle-related small molecules. Longitudinal sampling uncovered highly dynamic and unique building profiles that would be lost with WWTP-based sampling [23] or by sampling over longer time periods. For instance, the observation of significantly different features on weekdays versus weekends in multipurpose building 1 are likely reflective of a five-day workweek with a lower weekend occupancy, something not observed for the residential buildings. Along with building-specific information, we presented a series of methodological techniques that provide orthogonal and overlapping information for the analysis of longitudinal untargeted wastewater metabolomics. We highlight several main findings: the importance of temporal data collection, the utility of untargeted metabolomics for community monitoring, data-driven methods for information extraction, and the importance of direct, building sampling.

Temporal data acquisition, in combination with clustered feature modeling and time series comparisons, provides information not available from stationary statistical properties alone. We note that our sampling was at times sparse and irregular, a fact that hinders a more in depth analysis of patterns at various time scales; nevertheless, with the time series available it was still possible to observe benefits of longitudinal sampling. fTDs show that features may possess similar through-time mean intensities in different buildings, but with different temporal dynamics. These building-specific fluctuations may provide information on health-related events, given additional chemical identification. In light of the high level of feature intensity changes and subsequent mean reversion, it is likely that this study was not sampled at a high enough frequency for much of its duration. Additionally, replicate instrumental measurements were taken for only a few days; however, to better uncover feature intensity changes, multiple replicates at all time points are critical, as this lessens spurious intensity values from an already minimal wastewater sample that could represent a few individuals. As expected under the assumption that different individuals are generating waste within and across days, autocorrelation drops to near zero, even with a time lag of one, for the five largest clusters in each building. This observation indicates minimal information transfer between time points and agrees with the results of the Gaussian process modeling (S22 Fig). Frequent sampling may help explain select feature dynamics. For example, recreational drugs may be used at higher frequency on weekends rather than weekdays; thus, one might expect higher intensities on weekends. Further, the lack of differentiating features for Buildings 2 and 3 between weekdays and the weekend highlights the importance of temporal measurements; because large changes may be observed on any day, a statistical or aggregate approach would not properly capture these processes. This suggests that future studies should sample daily and—in certain circumstances—multiple times per day, requiring additional device engineering for fully automated sampling, unlike the current manual process. Finally, the importance of short, high-frequency sampling can be seen in our theoretical waste stream mixing analysis, which suggested that much of the dynamics information is lost with the addition of only a few additional waste streams.

An unanticipated finding from this temporal analysis was that numerous features display highly similar temporal dynamics within buildings. While many of these feature pairs correspond to different adducts or isotopes, a large number appear to be attributable to different metabolites, as their masses and retention times can differ by hundreds of Daltons and seconds (Figs 5C and 8 and S15 Fig). Specifically, focusing on highly correlated fTDs offers a means to discover new molecules (or features) linked to other molecules, events, or processes with known sets of features. Whether or not all of these features correspond to real metabolites, this information is readily supplied by fTDs and suggests perhaps non-obvious connections. In short, this approach may act as a hypothesis-generating method while also providing information about daily metabolite usage or exposure.

Untargeted metabolomics represents an information-rich method to monitor a community’s health and behavior. We putatively identified several human-related metabolites, most notably related to drugs, cosmetics, and food. For these, we observed that many features displayed similar intensities through time, but that their stability was frequently different across buildings. An unexpected observation was that most features possessed similar mean intensities between buildings, resulting in only a 12 being statistically significant with our stringent cutoff. Yet this small number appeared to provide important, building-specific information (Fig 2A). Such differentiating information may reflect the number of individuals using the toilet during the sampling period. While providing the potential for significant public health information, this method is limited by the ability to chemically identify each of the features observed. In addition, the use of only a single MS ionization mode and LC column type prevented a more complete report on the small molecule output of the buildings. These limitations warrant additional studies to expand feature-to-metabolite naming along with the use of select standards to validate putative metabolites.

A data-driven approach, based on classifying buildings using the features of a single day, recovers many of the features identified as important in other types of analysis, but also provides additional metabolites and features for follow up. Importantly, it extracts information relevant to each of the buildings in a minimally biased manner. The machine learning (ML) models we used found features in a manner complementary to the other presented methods, and demonstrate that it is possible to classify which building generated a specific, single-day waste profile. In addition to finding many of the 12 statistically significant stable features, the models also found features that belong to multiple classes of metabolite dynamics. For instance, 6-keto-decanoylcarnitine was important for building classification and was found to be unstable in Buildings 1 (Class 1) and 2 (Class 2), but stable in Building 3 (S5 Table). Our methods may prove useful for future temporal studies with the specific aim of public health monitoring. For instance, given data from well characterized control buildings and a new building of interest, using ML models and fTD clustering may help identify and track the dynamics of compounds in target communities.

Although direct building sampling was not the specific focus of this study, it was critical for this work, and our findings support the applicability of this technique for community-specific wastewater epidemiology. Most small-population studies have focused on targeted methods for measuring various drugs, with minimal temporal information [10,37]. Our recent 24-hour study likewise demonstrated the utility of upstream sampling, but of larger populations [23]. Single-building sampling minimizes the amount of time—and thus sample degradation—between sample generation and collection. This may address a potential source of uncertainty and error in WWTP-based measurements of population size or monitoring of illicit drug consumption [38–41]. Likewise, direct building sampling bypasses the issue of wastewater mixing or of occasional septage pumping into WWTPs, which may obfuscate fTDs or bias monitoring [1]. Thus, applications of the presented sampling method may include estimating population sizes and per capita feature values, and monitoring sporadic features.

High frequency, close-to-source sampling may, however, pose an ethical quandary. As the size of the population decreases, so does the anonymity of the results. For this reason, community-specific research must be conducted in such a way that personal health information remains confidential, and minimal negative consequences are experienced by the community under study [42].

## Material and methods

### Sample collection

Samples were collected from street-level manholes located outside of three buildings: one multipurpose-use building (Building 1), and two residential buildings (Buildings 2 and 3). We used a commercial peristaltic pump (Boxer) to continuously collect wastewater samples for 3 hours starting from 9:00 AM for Building 1 and 8:00 AM for Buildings 2 and 3. The peristaltic pump was programmed to pump wastewater at a rate of 5.55 mL/min over a 3-hour period into a 1 L polycarbonate bottle (Thermo Scientific) stored on ice, for a total volume of 1 L of wastewater. 100 mL of each sample were then filtered separately through a 0.2 μM PTFE membrane filter (Millipore) using a glass filtration apparatus (Glassco) to remove bacteria and debris. All filtration glassware and polycarbonate bottles were acid washed with hydrochloric acid and autoclaved prior to filtration. The filtrate was collected in amber glass vials, the pH was adjusted to between 2 and 3, and stored at -80 °C, all in less than 2 hours post sampling. Additional days for which only select building data was obtained were included only for intensity, stability and statistical analysis as well as classification and include: M_O2: B3, F_O13: B1/B2, Sa_O14: B1/B2, M_O16: B1/B3, T_O24: B2/B3, W_O25: B2/B3, W_N8: B1/B2, Sa_N11: B2/B3, W_N15: B3, Sa_N18: B2/B3, W_N29: B1, Sa_D9: B1/B2, W_D20: B1/B2.

### Liquid chromatography-mass spectrometry

10 μl of sample was analyzed via LCMS using a Vanquish ultra-performance liquid chromatography system coupled to an Orbitrap Fusion Lumos (Thermo Scientific) via a heated electrospray ionization (ESI) source. Data was collected in negative ionization mode with data-dependent secondary mass spectra (MS/MS) obtained via high-energy collisional dissociation (HCD, mass resolution 15,000 and collision energy of 35 arbitrary units, automatic gain control, AGT, of 5.0e4 and max injection time, IT, of 22 ms). The full MS resolution was 120,000 at 200 mz with an AGT target of 4.0e5 and a maximum IT of 50 ms. The quadrupole isolation width was set at 1.0 m/z. ESI was carried out at a source voltage of 2600 kV for negative ion mode with a capillary temperature of 350 °C, vaporizer temperature of 400 °C, and sheath, auxiliary, and sweep gases at 55, 20, and 1 arbitrary units, respectively.

Chromatographic separation was performed on a Waters Acquity HSS T3 column (2.1 × 100 mm, 1.8 μm) equipped with a Vanguard pre-column and maintained at 40 °C. The column was eluted with (A) 0.1% formic acid in water and (B) 0.1% formic acid in acetonitrile at a flow rate of 0.5 mL min^-1^. The gradient started at 1% B for 1 min, ramped to 15% B from 1–3 min, ramped to 50% from 3–6 min, ramped to 95% B from 6–9 min, held until 10 min, ramped to 1% from 10–10.2 min, and finally held at 1% B (total gradient time 12 min). Run order was randomized over two batches of samples with pooled quality control samples run intermittently (every 6 or 7 samples) along with MilliQ water blanks to account for the general background of solvent system and mass spectrometer.

### Data processing

Python 3.6.5 with scikit-learn version 0.19.1 as well as R 3.5.1 were used for processing and analysis. Following data acquisition, all data files were converted to an open source file format (.mzML) via a custom wrapper (msconvert_ee.py) of the program MSConvert in the ProteoWizard suite [43]. All files were then processed as a single batch with a custom python wrapper script (full_ipo_xcms.py) of both IPO [44] and then subsequent XCMS [45] processing. The parameters for XCMS were: CentWave (ppm = 10, peakwidth = (5,15), snthresh = (100), prefilter = (4,10000), mzCenterFun = wMean, integrate = 2, mzdiff = -0.005, noise = 50,000), ObiwarpParam (binsize = 0.1, response = 1, distFun = cor_opt, gapInit = 0.3, gapExtend = 2.4, factorDiag = 2, factorGap = 1), PeakDensityParam (bw = 10, minFraction = 0.05, minSamples = 1, binSize = 0.002, maxFeatures = 50), mode (negative). In addition to aligning and extracting peak information, this program automatically extracted all MS/MS spectra and saved as a separate .mgf file for use in the metabolite naming pipeline.

All features were binary log transformed, after which the standard deviation was calculated. Features with pooled sample coefficient of variance in excess of 0.3 were removed from further analysis. The data also was corrected for run order using the local (two closest run order-flanking quality control, QC, samples) and global (all) QC feature values where the normalized feature intensity was calculated with the following formula:

X′=X*(R/C) (1)

Here *X*′ corresponds to the corrected value, *X* the input feature value, *R* the global average of the feature over all QC sample and *C* the local feature QC average. All samples were corrected in this way.

All samples were blank subtracted (mean blank intensity for each feature) and resulting values less than 0 or missing values were filled with one half the minimum of the feature’s intensity in a given building. Only features for which the cross building sum of log intensities was greater than 100 (S1 Text) were kept and days with replicates were averaged (B1: O4, O18, O21, N4, D2, D20; B2: O4, O18, O21, N4, D2, D20; B3: O4, O18, O20, O21, N4).

### Putative metabolite identification

For ease of figure presentation when named features or lists thereof exceeded defined sizes, the name was replaced with its HMDB chemical subclass or direct parent for which each mz-rt tuple can be mapped to names in the corresponding supplementary table. Given only putative identification throughout, all names should be interpreted with caution.

Identification was automated using custom python scripts, outlined in the supporting information. It performed a primary mass-to-charge look up of the exact mass accounting for multiple possible adducts and isotopes ([M]^-^, [M-H]^-^, [M+Cl^-^]^-^, [M-H-H_2_O]^-^, [2M-H]^-^, [M-2H+Na^+^]^-^, [M-2H+K^+^]^-^, [M+(1–3)^13^C-H]^-^) in four databases: MetaCyc [46], the Human Metabolite DataBase (HMDB) [47], the Chemical Entities of Biological Interest (ChEBI) [48], and LIPID MAPS [49]. For this lookup, we report putative chemical names if the parts per million (ppm) error was ≤ 5, this represented an identification level of 3. Following this matching, the named features with associated ppm error and adducts or isotopes were ranked according to a heuristic order of likelihood of being observed (see S1 Text for ordering). Given equally ranked adducts or isotopes, priority was given to the chemicals with the lowest ppm error. The second part of the naming was to perform *in silico* fragmentation matching using MetFrag [50,51] on all ions with secondary mass spectra (MS2) that were extracted during the initial XCMS feature extraction. For this, individual MetFrag programs were run in parallel on MS2 scans (on up to three different MS2 spectra for the precursor ion), for all of the following parent ion possibilities: [M]^-^, [M-H]^-^, [M+Cl^-^]^-^, [M-H-H_2_O]^-^, [2M-H]^-^, [M-2H+Na^+^]^-^, and [M-2H+K^+^]^-^. The MetFrag outputs for each parent ion were combined and ranked in order of MetFrag probability, these names were then matched to the primary mass-named metabolites with matches being given the highest ranking in terms of metabolite identification (level 2). Following naming, some putative labels were removed, specifically those with select elements, polymers or ‘R-groups’ (S1 Text and S1 Table).

### Chemical class mapping

Each named feature was matched to its corresponding feature in one of the following parsed databases: HMDB, LIPID MAPS, ChEBI or MetaCyc. From this initial search, if not in HMDB, the name, Inchi string, InchiKey, HMDB ID and HMDB accession numbers were extracted if available for the compound. These were each used to look up the compound in the HMDB database from which class, subclass and direct parent values were obtained. If no label was available at the specific taxonomic level, the next available, higher class name was used. Chemical taxonomy from different databases was not mixed. If no class was found for any putative names, the feature was counted as having a putative annotation but no classification (‘putative annotation no class’ label in plots). The only exception is the isoquinoline and sulfonamide in Fig 2A for which the closest parent node in the ChEBI ontology was used as they could not be found in HMDB.

### Statistical analysis

Multiple comparison tests of feature intensity differences between more than two buildings were done with the non-parametric Kruskal-Wallis test followed by Benjamini-Hochberg FDR correction (scipy.stats.kruskal, statsmodels.stats.multitest.multipletests). For stationary statistical significance an FDR corrected P-value of less than 0.00001 was used. Linear regression on mean feature values and the associated correlation constants were extracted using scipy.stats.linregress.

### Feature time series summary statistics

Through-time mean log intensity and standard deviation values were calculated for all features in each building individually. Features with a standard deviation ≥ 2 were labeled unstable, and stable otherwise. Given these labels, building overlap analysis was performed using venn3 and venn3_circles (matplotlib_venn) for which all building overlaps were retained. To determine feature color in Fig 1D, a feature was considered to be stable between the compared buildings if it was stable in either (colored blue).

### Temporal feature profile analysis—building clustering

All features were z-normalized through time (temporal mean subtracted and divided by the standard deviation) and each building was clustered by K-means clustering (sklearn.cluster.KMeans) using 100 clusters with all other parameters kept to their default values. Each feature was mapped to its corresponding mz and rt value for plotting as well as being grouped by cluster size. Mean cluster values were extracted for each of the clusters and used to train a GP regression model (sklearn.gaussian_process.GaussianProcessRegressor, alpha = 0.0001) using a radial basis function kernel with a 0.125 length_scale parameter mixed linearly with a constant kernel with noise corresponding to the mean standard deviation of elements of the cluster (sklearn.gaussian_process.kernels.RBF and .ConstantKernel respectively).

### Temporal feature profile analysis—inter and intra building feature analysis

Time series distances were calculated via a Euclidean distance metric (scipy.spatial.distance.euclidean). All-to-all feature distance matrix calculation within and between buildings was performed using scipy.spatial.distance_matrix while the one-to-one distance of each feature to the corresponding feature in the other building using the euclidean function. For the all-to-all, feature pairs that met the similarity cutoff of either 1.5 or 2.82 were kept and further analyzed for both rt and mz difference between features. Pearson correlations were calculated using the .corr() method of Pandas DataFrame objects. Ward hierarchical clustering was performed using the linkage function from scipy.cluster.hierarchy.

### Machine learning model training and analysis

L1-LR and RF models were built to predict which building a single day-feature profile belonged to (sklearn.ensemble.RandomForestClassifier, and sklearn.linear_model.LogisticRegressionCV with the ‘saga’ solver). To gain statistics on model performance, each was trained 50 times on random, full data shuffles (sklearn.utils.shuffle). For each data shuffle, the days were randomly split 75:25 into train and test splits respectively. The log ion intensity data training split was standardized (sklearn.preprocessing.StandardScaler), and the test data transformed. Internal cross validation (3-fold) on the training split was performed internal to the LogisticRegressionCV class while the number of trees for the RF was set to 1000 requiring no cross validation. Following training, all models were evaluated on the fully held out test set. ROC-AUC analysis was performed for each separate model (using sklearn.metrics.roc_curve and .auc) during the testing phase. For the 50 models built, the feature coefficients of the L1-LR models or the feature importances from the RF were averaged. To isolate unique building features, averaged features that had non-zero feature coefficients in at least 40 of the L1-LR models and an averaged feature importance in the RF models of > 0.005 were extracted.

### ‘Important’ feature and cross building analysis

Temporal similarity values were calculated between all features to the IFs (all features from S2–S5 Tables) in each building separately using a Euclidean distance metric. Many features possessed a distance < 1.5 to multiple IFs and were counted for the size of each IF’s group. After calculating the size of each group, all features were then only assigned to the largest group they belonged to and those with sizes greater than 20 for Building 1 and 2 or 5 for Building 3 were input to metric MDS (sklearn.manifold.MDS).

To find features that co-clustered between buildings, the cluster memberships for all buildings were fully compared, including between all three buildings. If the set of features in the intersection of either 2 or 3 clusters (each from different buildings) was > 5, the cluster pair overlap of features was kept. A cluster pairing was only further analyzed if the difference in minimum and maximum rt was greater than 30 s.

## Supporting information

S1 Table Name components that caused a label to be discarded. If any of the following showed up in the name or chemical formula for a putative name for a feature, it was removed from consideration as a plausible name.
(XLSX)

S2 Table mz, rt and putative name for each stable, but statistically significant feature. Features map to Fig 2A. **denotes level 3 ID, otherwise, if named, it is a level 2 ID. For level 2 IDs only the chemical name and MetFrag probability are presented, for level 3 there is the name, ppm error, adduct and database matched and chemical formula.
(XLSX)

S3 Table. mz , rt and putative name for select metabolites of each temporal dynamics class. All IDs are level 2 with name and MetFrag probability. Only level 2 putatively named features were chosen and are used only as examples of the characteristics of the dynamics class.
(XLSX)

S4 Table mz, rt and putative names for select metabolite classes. **denotes level 3 ID, otherwise if a name is supplied it is a level 2 ID. For level 2 IDs only the chemical name and MetFrag probability are present, for level 3 there is the name, ppm error, adduct and database matched and chemical formula.
(XLSX)

S5 Table Features important from both the RF and LR models. Features with ‘**’ denote that they are from solely primary mz database name matching (minimum reporting standards level 3) and where possible show a name, ppm error, which database and adduct was used and chemical formula. Features with solely a name and subsequent number are from mz-to-database match along with Metfrag identification.
(XLSX)

S6 Table Unnormalized MS data for all days and buildings. (XLSX)

S7 Table Z-normalized MS data for all days and buildings. (XLSX)

S8 Table Feature summary. Including mz, rt, ionization mode, charge-correction, compound ID used, stability type in each building, classifier use and K-means cluster membership.
(XLSX)

S9 Table MS/MS fragmentation data for matched compounds. (XLSX)

S1 Fig Building breakdown of stable and unstable features with further unstable feature subclassification. (Top row) Histogram of feature through-time standard deviation, thick dashed vertical line at 2 represents the cuboff between stable and unstable features. Light, dotted vertical line at 3.5 represents the cutoff for which features greater than were assigned to unstable class 2. (Bottom row) Histogram of median feature intensity for the remaining unstable features (class 2 removed). Vertical line at 14 demarcates class 1 from class 3 unstable features.
(PDF)

S2 Fig Stable and unstable features are primarily unannotated but many, low count classes, subclasses and direct parents can be observed. Shown for each are the top 20 categories by size.
(PDF)

S3 Fig Statistical analysis between weekdays and the weekend with chemical class breakdowns of the Building 1 significant features. (A) Q-value analysis of Wednesday versus Saturday. (B) Q-value analysis of weekdays versus weekends. (C) Chemical class, subclass and direct parent of each of the Q<0.05 features from building 1 in part B. Each plot maximally shows the 20 most abundant classes.
(PDF)

S4 Fig Log Q-value histograms with select cutoff for all features, stable features and unstable features and chemical class of significant features using all features. The following are the 38 lowest log Q-value features from ‘all features’ (top left plot): 1) 2-hydroxyethanesulfonate, 1.0; 2) (2-hydroxyethoxy)sulfonic acid**, 0.482, M-H_hmdb, C2H6O5S1; 3) l-2,3-dihydrodipicolinate, 1.0, 2-furoylglycine, 1.0; 4) n-acetyl-5-aminosalicylic acid, 1.0, 6-hydroxy-3-succinoylpyridine, 1.0; 5) pantothenol, 1.0; 6) threoninyl-proline, 1.0, gamma-glutamyl-gamma-aminobutyraldehyde, 1.0; 7) l-iditol, 1.0, d-altritol, 1.0; 8) 219.04563Da/33.92086s; 9) n-decanoylglycine, 1.0; 10) 237.93104Da/209.2619s; 11) girgensonine, 1.0; 12) (4R)-4,8-dimethylnonyl hydrogen sulfate**, 0.690, M-H_chebi, C11H24O4S1; 13) n-lauroylglycine, 1.0; 14) 4’-dihydroabscisic acid, 0.8915065391457075; 15) 267.14393Da/545.57034; 16) ferulic acid 4-sulfate, 1.0, isoferulic acid 3-sulfate, 1.0; 17) felbamate, 1.0; 18) 1,4,5-trihydroxy-&Delta;^2,3^-protoilludene**, 4.840, M-2H_Na_meta, C15H24O3, illudol**, 4.840, M-2H_Na_meta, C15H24O3, isotrichodiol**, 4.840, M-2H_Na_meta, C15H24O3, 3-hydroxylubimin**, 4.840, M-2H_Na_meta, C15H24O3, 1-Hydroxyepiacorone**, 4.840, M-2H_Na_hmdb, C15H24O3, Acorusdiol**, 4.840, M-2H_Na_hmdb’, C15H24O3, 3(4-&gt;5)-Abeo-4,11:4,12-diepoxy-3-eudesmanol**, 4.840, M-2H_Na_hmdb, C15H24O3, (4R,5S,7R,11R)-11,12-Dihydroxy-1(10)-spirovetiven-2-one**, 4.840, M-2H_Na_hmdb, C15H24O3, Apotrichodiol**, 4.840, M-2H_Na_hmdb, C15H24O3, 6alpha-Carissanol**, 4.840, M-2H_Na_hmdb, C15H24O3, alpha-Carissanol**, 4.840, M-2H_Na_hmdb, C15H24O3, Epioxylubimin**, 4.840, M-2H_Na_hmdb, C15H24O3, Dihydromyoporone**, 4.840, M-2H_Na_hmdb, C15H24O3, Piperalol**, 4.840, M-2H_Na_hmdb, C15H24O3, Zedoarondiol**, 4.840, M-2H_Na_hmdb, C15H24O3, Hydroxypelenolide**, 4.840’, M-2H_Na_hmdb’, C15H24O3, Toxin FS2**, 4.840, M-2H_Na_hmdb, C15H24O3, Urodiolenone**, 4.840, M-2H_Na_hmdb, C15H24O3, Bisacurone B**, 4.840, M-2H_Na_hmdb, C15H24O3, 2,3-Dihydroabscisic alcohol**, 4.840, M-2H_Na_hmdb, C15H24O3, 3-Methyl-5-pentyl-2-furanpentanoic acid**, 4.840, M-2H_Na_hmdb, C15H24O3, 3-Methyl-5-propyl-2-furanheptanoic acid**, 4.840, M-2H_Na_hmdb, C15H24O3, 9-(3,5-dimethylfuran-2-yl)-nonanoic acid**, 4.842, M-2H_Na_lipid, C15H24O3, 3-methyl-5-pentyl-2-furanpentanoic acid**, 4.842, M-2H_Na_lipid, C15H24O3, Dihydromyoporone**, 4.842, M-2H_Na_lipid, C15H24O3, (+)-2-Sterpurene-6,12,15-triol**, 4.842, M-2H_Na_lipid, C15H24O3, 9-Hydroxy-helminthosporol**, 4.842, M-2H_Na_lipid, C15H24O3, Dendrobane A**, 4.842, M-2H_Na_lipid, C15H24O3; 19) 1-(3-chlorophenyl)-4-hexylpiperazine**, 0.128, M-H_chebi, C16H25Cl1N2; 20) 294.89324Da/28.30617s; 21) ropinirole, 1.0; 22) 4’-n-desmethylolanzapine, 1.0; 23) threoninyl-tyrosine, 1.0, tyrosyl-threonine, 1.0; 24) 320.19064Da/475.77286; 25) acetohexamide, 1.0; 26) 6-keto-decanoylcarnitine, 1.0; 27) 331.22431Da/330.17615s; 28) amifloxacin, 1.0; 29) 5(s)-hydroperoxyeicosatetraenoic acid, 1.0, 15(s)-hpete, 1.0, lmfa03060044, 1.0, lmfa03060045, 1.0, 12(r)-hpete, 1.0, 11(r)-hpete, 1.0, 9s-hpete, 1.0), (’8(s)-hpete’, 1.0), 12(r)-hpete, 1.0, 11(r)-hpete, 1.0, 8(s)-hpete, 1.0, 5-hpete, 1.0, 15-hpete, 1.0, 5-hpete, 1.0, 15(s)-hpete, 1.0, 9(s)-hpete, 1.0; 30) 7s,8s-dihode, 1.0, 5s,8r-dihode, 1.0, (7s,8s)-dihode, 1.0, 15,16-dihode, 1.0, (±)-(e)-13-hydroxy-10-oxo-11-octadecenoic acid, 1.0; 31) 359.25568Da/381.54309; 32) Zanthobisquinolone**, 4.035, M-H_hmdb, C21H18N2O4; 33) 2,6-dideoxy-4-O-methyl-alpha-D-arabino-hexopyranosyl-(1->3)-4-O-acetyl-2-O-methyl-alpha-L-fucopyranose**, 2.268, M-H_chebi, C16H28O9; 34) 365.07355Da/545.96533s; 35) 7-hydroxyflavanone 7-O-beta-D-glucoside**, 1.452, M-H_chebi, C21H22O8, (3’R,4’R)-3’-Epoxyangeloyloxy-4’-acetoxy-3’,4’-dihydroseselin**, 1.452, M-H_chebi, C21H22O8, nobiletin**, 1.452, M-H_chebi, C21H22O8, graminone B**, 1.452, M-H_chebi, C21H22O8, 2-(2,5-dimethoxyphenyl)-5,6,7,8-tetramethoxy-4H-1-benzopyran-4-one**, 1.452, M-H_chebi, C21H22O8, 2-(3,5-dimethoxyphenyl)-5,6,7,8-tetramethoxy-4H-1-benzopyran-4-one**, 1.452, M-H_chebi, C21H22O8, hexamethylquercetagetin**, 1.452, M-H_chebi, C21H22O8, 5-[(5-methoxycarbonyl-2-methyl-3-furanyl)methoxy]-2-methyl-3-benzofurancarboxylic acid 2-methoxyethyl ester**, 1.452, M-H_chebi, C21H22O8, 5,6,7-trimethoxy-3-(3,4,5-trimethoxyphenyl)-1-benzopyran-4-one**, 1.452, M-H_chebi, C21H22O8; 36) didemethylasterriquinone D**, 3.542, Cl_meta, C22H14N2O4; 37) 2-[[2-[2-(2,3-dihydroindol-1-yl)-2-oxoethyl]-1-oxo-5-isoquinolinyl]oxy]acetic acid ethyl ester**, 3.826, M-H_chebi, C23H22N2O5; 38) 489.20314Da/226.67616. **level 3 identification.
(PDF)

S5 Fig The three unstable feature groups are predominantly unannotated at the chemical class, subclass and direct parent levels. (PDF)

S6 Fig Building 1 chemical classification of the features in the top five largest clusters. Each row depicts a single cluster with the columns corresponding to class, sub class and direct parent. Each plot maximally shows the 20 most abundant classes.
(PDF)

S7 Fig Building 2 chemical classification of the features in the top five largest clusters. Each row depicts a single cluster with the columns corresponding to class, sub class and direct parent. Each plot maximally shows the 20 most abundant classes.
(PDF)

S8 Fig Building 3 chemical classification of the features in the top five largest clusters. Each row depicts a single cluster with the columns corresponding to class, sub class and direct parent. Each plot maximally shows the 20 most abundant classes.
(PDF)

S9 Fig Large feature deviations from the mean are lost with the addition of relatively few waste streams. (A–E) Mixing of building waste with 0–50 additional simulated waste streams, with clustering and GP fitting. The two largest clusters are plotted, with their standard deviations in gray. (F) Plot of the sum of Euclidean distances between all pairs of cluster centers for a given number of mixed buildings. Error bars represent the standard deviation following 10 repeats.
(PDF)

S10 Fig Feature dynamics for possible glucoside-related metabolites. Compounds with ‘glucosid’ in their name, whether matched at level 2 or 3 were included in the plot. S4 Table for complete details.*hinokitiol glucoside / (3r,4r)-4,8-dihydroxy-3-((r)-2-hydroxypentyl)-6,7-dimethoxyisochroman-1-one, **methyl (3x,10r)-dihydroxy-11-dodecene-6,8-diynoate 10-glucoside / methyl 3,4-dihydroxy-5-prenylbenzoate 3-glucoside, ***5,6,7-trimethoxy-3-(3,4,5-trimethoxyphenyl)-1-benzopyran-4-one / (3’r,4’r)-3’-epoxyangeloyloxy-4’-acetoxy-3’,4’-dihydroseselin / 5-[(5-methoxycarbonyl-2-methyl-3-furanyl)methoxy]-2-methyl-3-benzofurancarboxylic acid 2-methoxyethylester/nobiletin/graminone b / hexamethylquercetagetin / 2-(2,5-dimethoxyphenyl)-5,6,7,8-tetramethoxy-4h-1-benzopyran-4-one / 7-hydroxyflavanone 7-o-beta-d-glucoside / 2-(3,5-dimethoxyphenyl)-5,6,7,8-tetramethoxy-4h-1-benzopyran-4-one, ****quercetin 3-o-alpha-l-[6‴-p-coumaroyl-beta-d-glucopyranosyl-(1->2)-rhamnopyranoside]-7-o-beta-d-glucopyranoside / kaempferol 3-o-[6-(4-coumaroyl)-beta-d-glucosyl-(1->2)-beta-d-glucosyl-(1->2)-beta-d-glucoside].
(PDF)

S11 Fig Intrabuilding Euclidean feature pair distance histograms and percent of total pairs covered as a function of distance. (A and B) Building 1 plots. (C and D) Building 2 plots. (E and F) Building 3 plots. For all plots, vertical black lines are drawn at select distance values as indicated.
(PDF)

S12 Fig Interbuilding Euclidean feature pair distance histograms and percent of total pairs covered as a function of distance. (A and B) Buildings 1–2 plots. (C and D) Buildings 1–3 plots. (E and F) Buildings 2–3 plots. For all plots, vertical black lines are drawn at select distance values as indicated.
(PDF)

S13 Fig Removing all temporally related feature pairs with at least one possessing a through-time mean intensity less than minimum intensity value (distance cutoff = 2.82 for similarity). (PDF)

S14 Fig Removing all temporally related feature pairs with at least one possessing a through-time mean intensity less than minimum intensity value (distance cutoff = 1.5 for similarity). (PDF)

S15 Fig Complete delta mz, rt 2-D histogram of temporally similar features for the three buildings at different distances. (A) Full mz and rt domain for < 1.5 distance features. (B) Reduced and (C) full domain histograms for features with similarity < 2.82 in Euclidean distance.
(PDF)

S16 Fig Complete 2D-histogram of Pearson correlations versus the difference in feature pair mz values for all three buildings. (PDF)

S17 Fig Anticorrelated feature pairs (< -0.06) with non-statistically different intensity distributions (Q > 0.05) and examples for each building. (A) Building 1 (B) Building 2, *caffeic acid 4-sulfate alternative compound. (C) Building 3 feature pairs along with example feature time course. For all time series the top label corresponds to the blue time series while the red is the second entry. ‘Mean’ refers to a feature’s through-time mean intensity with standard deviation.
(PDF)

S18 Fig Anticorrelated features (< -0.6) between building pairs. Labeled features are level 2 IDs unless marked with a ^ which corresponds to level 3. The number below the names is the feature’s building-to-building correlation value.
(PDF)

S19 Fig Receiver operating characteristic (ROC) area under the curve (AUC) analysis for the random forest (RF) models. Mean of the three AUCs presented as the overall AUC.
(PDF)

S20 Fig Chemical classification of top machine learning discovered features. Each plot maximally shows the 20 most abundant classes.
(PDF)

S21 Fig Co-clustered features between buildings with at least 5 features shared in the clusters between the buildings and a minimum delta intracluster rt min and max of 30 s. Each color corresponds to co-clustered features between the compared buildings. Co-clustering does not indicate shared dynamics between buildings, only that the features had similar dynamics internal to each building.
(PDF)

S22 Fig Autocorrelation analysis of the 5 largest clusters for each of the 3 buildings showing rapid loss of autocorrelation. Autocorrelation was calculated at all lag times for the data set Ticks show the first and last sampling dates of each month (October, November, December). Autocorrelation was calculated with statsmodels.tsa.stattools.acf.
(PDF)

S23 Fig Feature intensities of B1, D16 relative to mean feature intensities for the other days in B1. (PDF)

S24 Fig Elbow plots for the cluster inertia as a function of the number of clusters used for K-means clustering. (PDF)

S25 Fig Enrichment of features used relative to the number of clusters. The clusters were first sorted by size and then for each number of clusters, the number of cluster members was summed for that value of combined clusters.
(PDF)

S26 Fig Histograms of feature pair differences in retention time. (A) All feature pairs with differences in rt of > 30s showing select delta rt bins possessing increased counts. (B) Same as in A but with a second axis of the mz differences depicting select columns with higher counts than neighboring columns.
(PDF)

S1 Text Additional data processing methods. Detailed methods, additional text and commands for reproducing this work using associated code and data.
(DOCX)
