# Supplementary figures and images for: Longitudinal wastewater sampling in buildings reveals temporal dynamics of metabolites
Source: PLoS Comput Biol. 2020 Jun 29;16(6):e1008001. doi: 10.1371/journal.pcbi.1008001 (PMC7351223; doi:10.1371/journal.pcbi.1008001)

Building 1

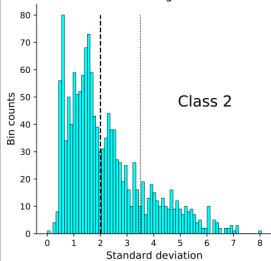

Building 2

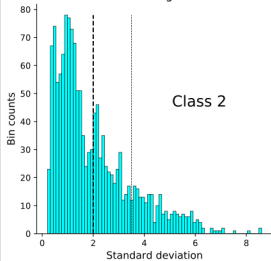

Building 3

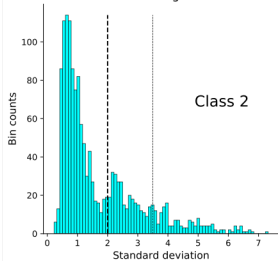

B1

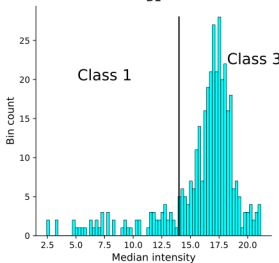

B2

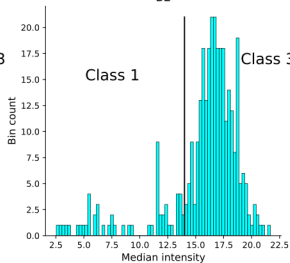

B3

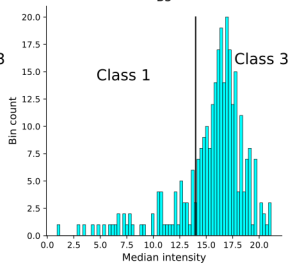

Supplement: S1 Fig — (Top row) Histogram of feature through-time standard deviation, thick dashed vertical line at 2 represents the cuboff between stable and unstable features. Light, dotted vertical line at 3.5 represents the cutoff for which features greater than were assigned to unstable class 2. (Bottom row) Histogram of median feature intensity for the remaining unstable features (class 2 removed). Vertical line at 14 demarcates class 1 from class 3 unstable features. (PDF) [file pcbi.1008001.s010.pdf]

A

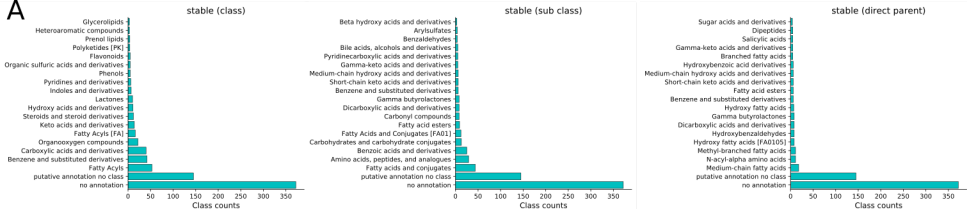

B

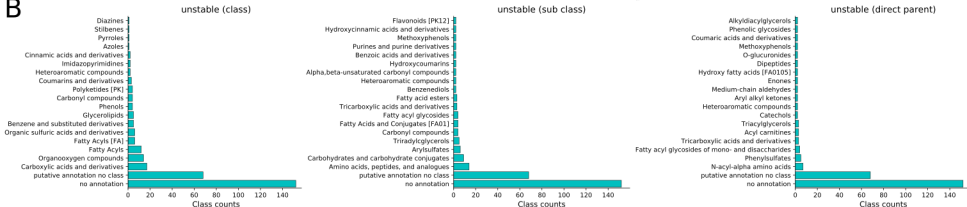

Supplement: S2 Fig — Shown for each are the top 20 categories by size. (PDF) [file pcbi.1008001.s011.pdf]

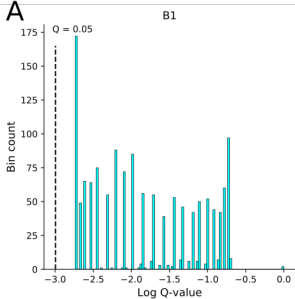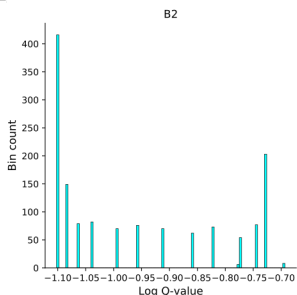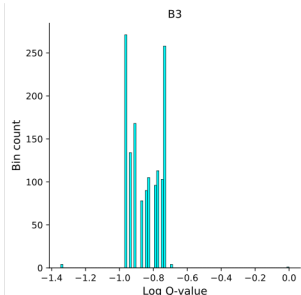

Week v. weekend

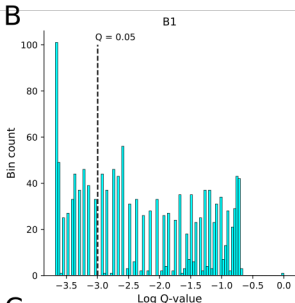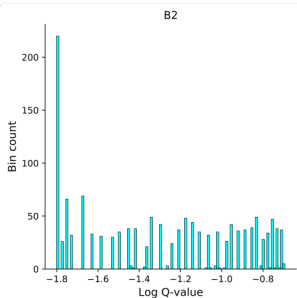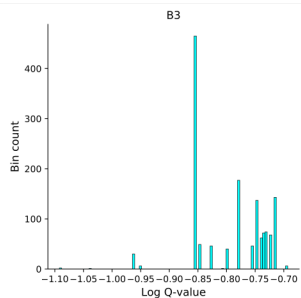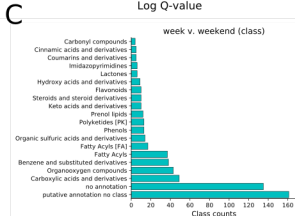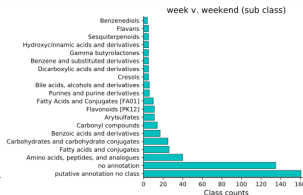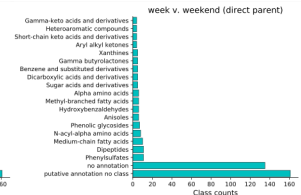

Supplement: S3 Fig — (A) Q-value analysis of Wednesday versus Saturday. (B) Q-value analysis of weekdays versus weekends. (C) Chemical class, subclass and direct parent of each of the Q<0.05 features from building 1 in part B. Each plot maximally shows the 20 most abundant classes. (PDF) [file pcbi.1008001.s012.pdf]

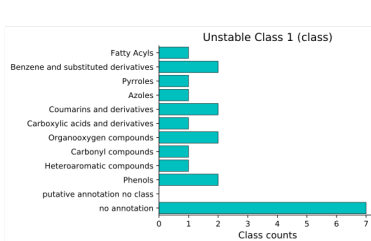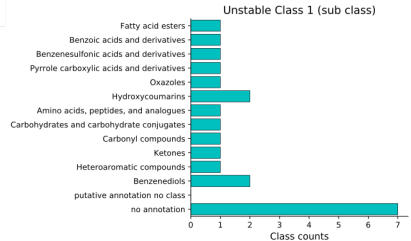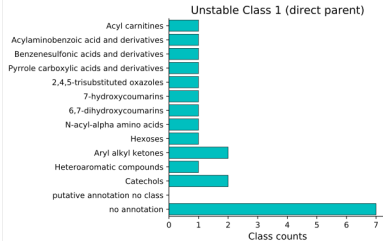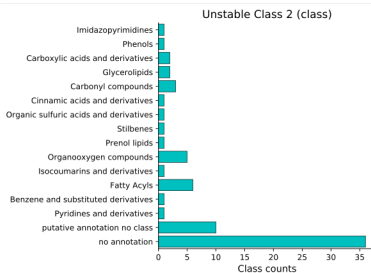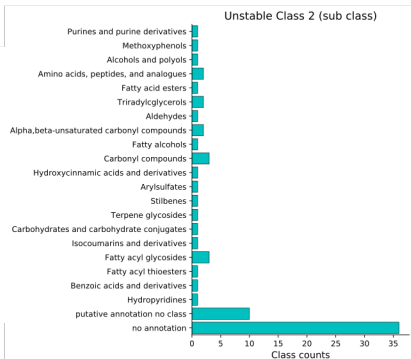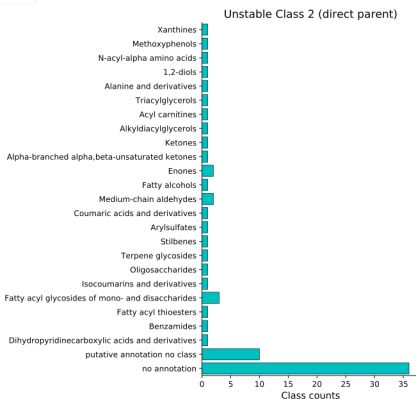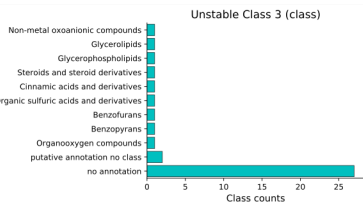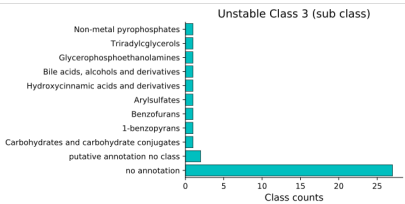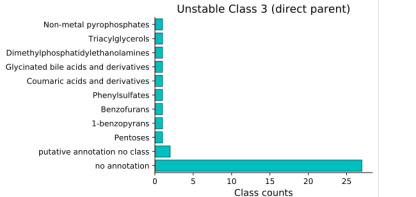

Supplement: S5 Fig — (PDF) [file pcbi.1008001.s014.pdf]

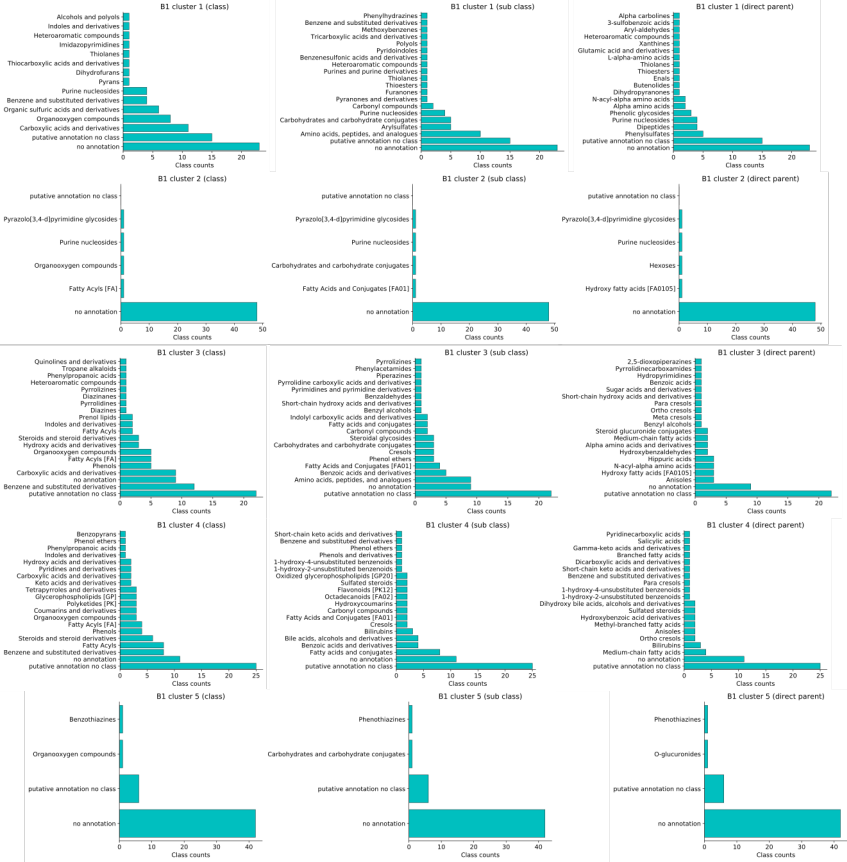

Supplement: S6 Fig — Each row depicts a single cluster with the columns corresponding to class, sub class and direct parent. Each plot maximally shows the 20 most abundant classes. (PDF) [file pcbi.1008001.s015.pdf]

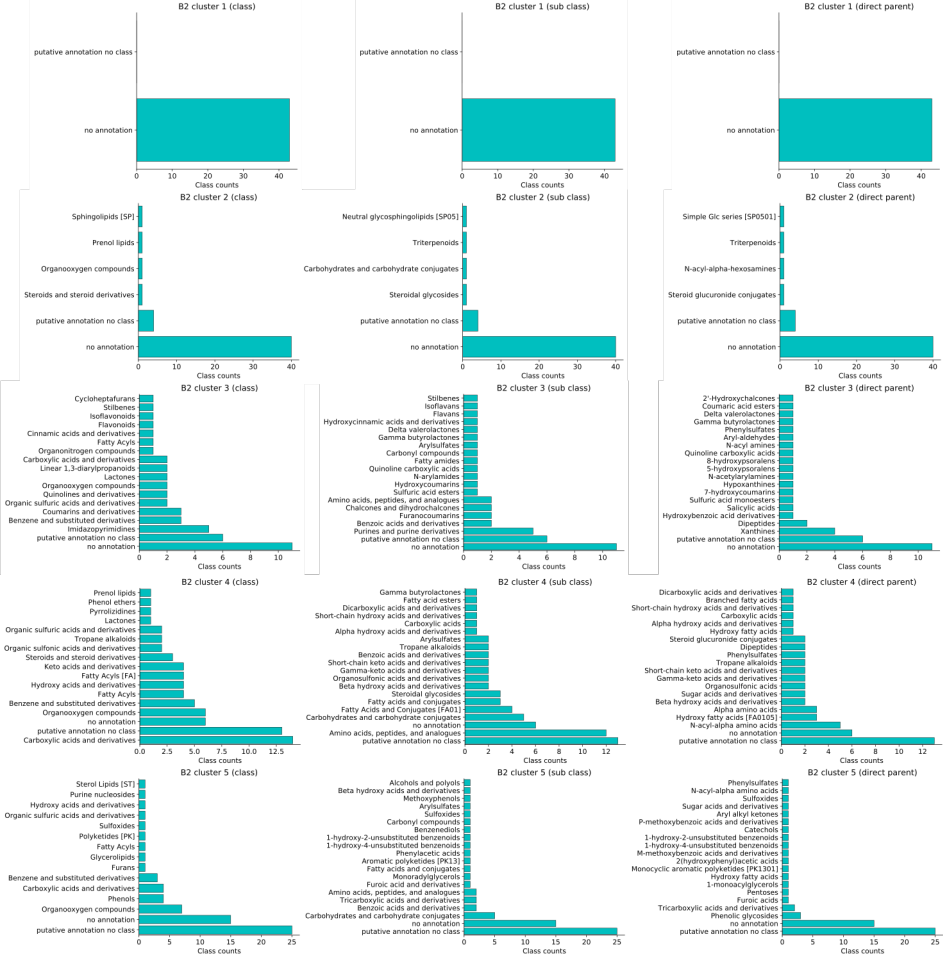

Supplement: S7 Fig — Each row depicts a single cluster with the columns corresponding to class, sub class and direct parent. Each plot maximally shows the 20 most abundant classes. (PDF) [file pcbi.1008001.s016.pdf]

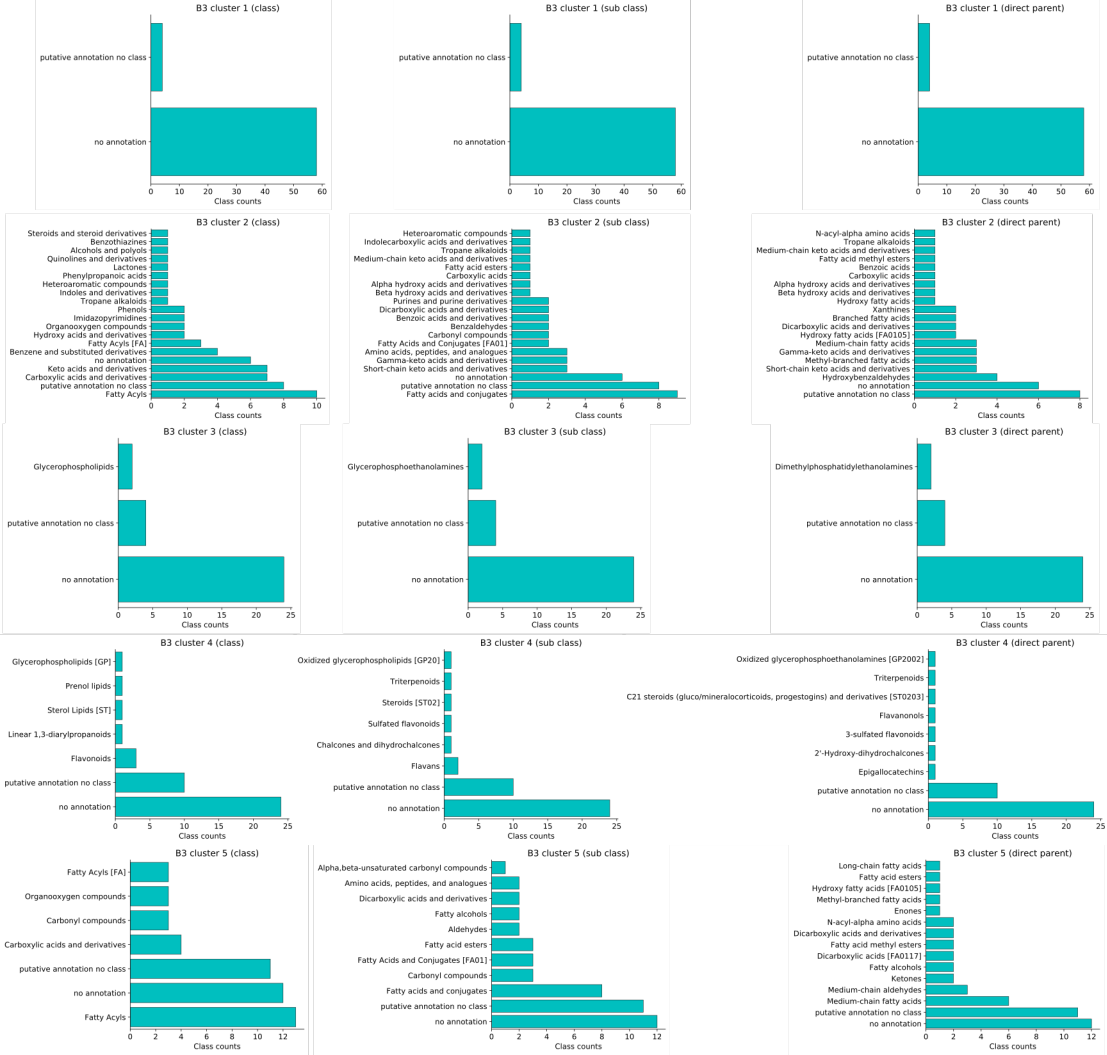

Supplement: S8 Fig — Each row depicts a single cluster with the columns corresponding to class, sub class and direct parent. Each plot maximally shows the 20 most abundant classes. (PDF) [file pcbi.1008001.s017.pdf]

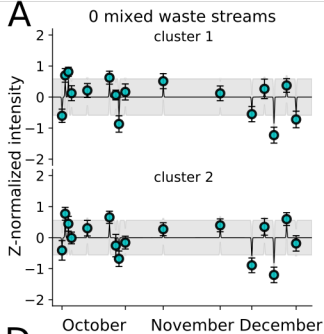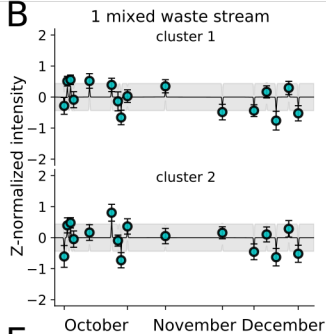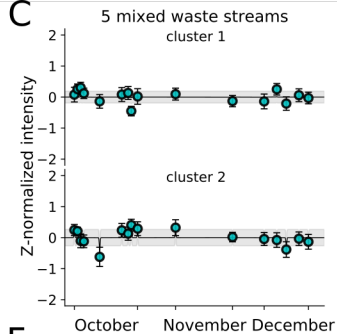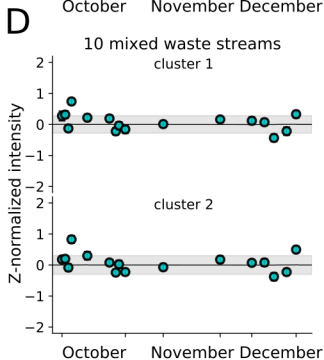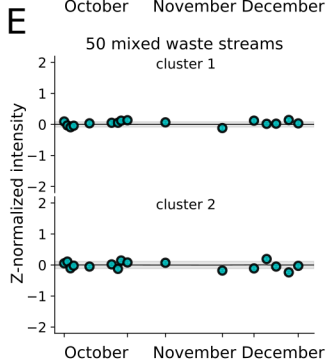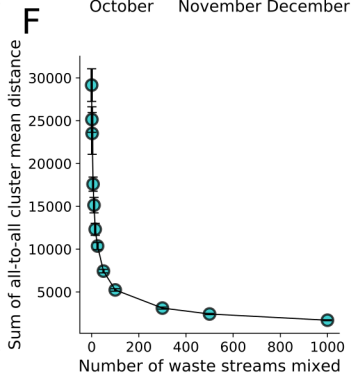

Supplement: S9 Fig — (A–E) Mixing of building waste with 0–50 additional simulated waste streams, with clustering and GP fitting. The two largest clusters are plotted, with their standard deviations in gray. (F) Plot of the sum of Euclidean distances between all pairs of cluster centers for a given number of mixed buildings. Error bars represent the standard deviation following 10 repeats. (PDF) [file pcbi.1008001.s018.pdf]

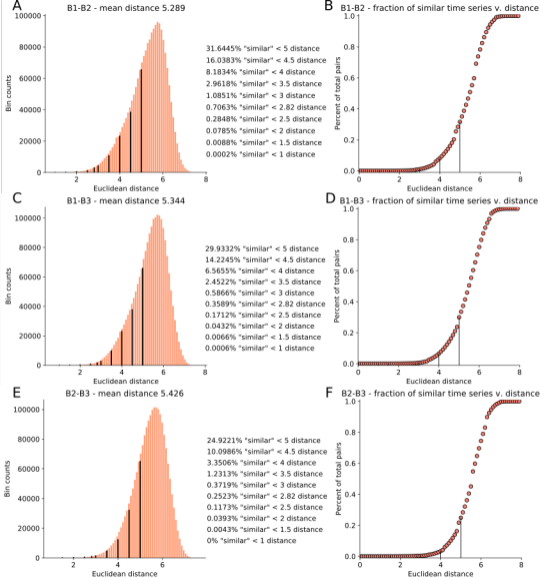

Supplement: S12 Fig — (A and B) Buildings 1–2 plots. (C and D) Buildings 1–3 plots. (E and F) Buildings 2–3 plots. For all plots, vertical black lines are drawn at select distance values as indicated. (PDF) [file pcbi.1008001.s021.pdf]

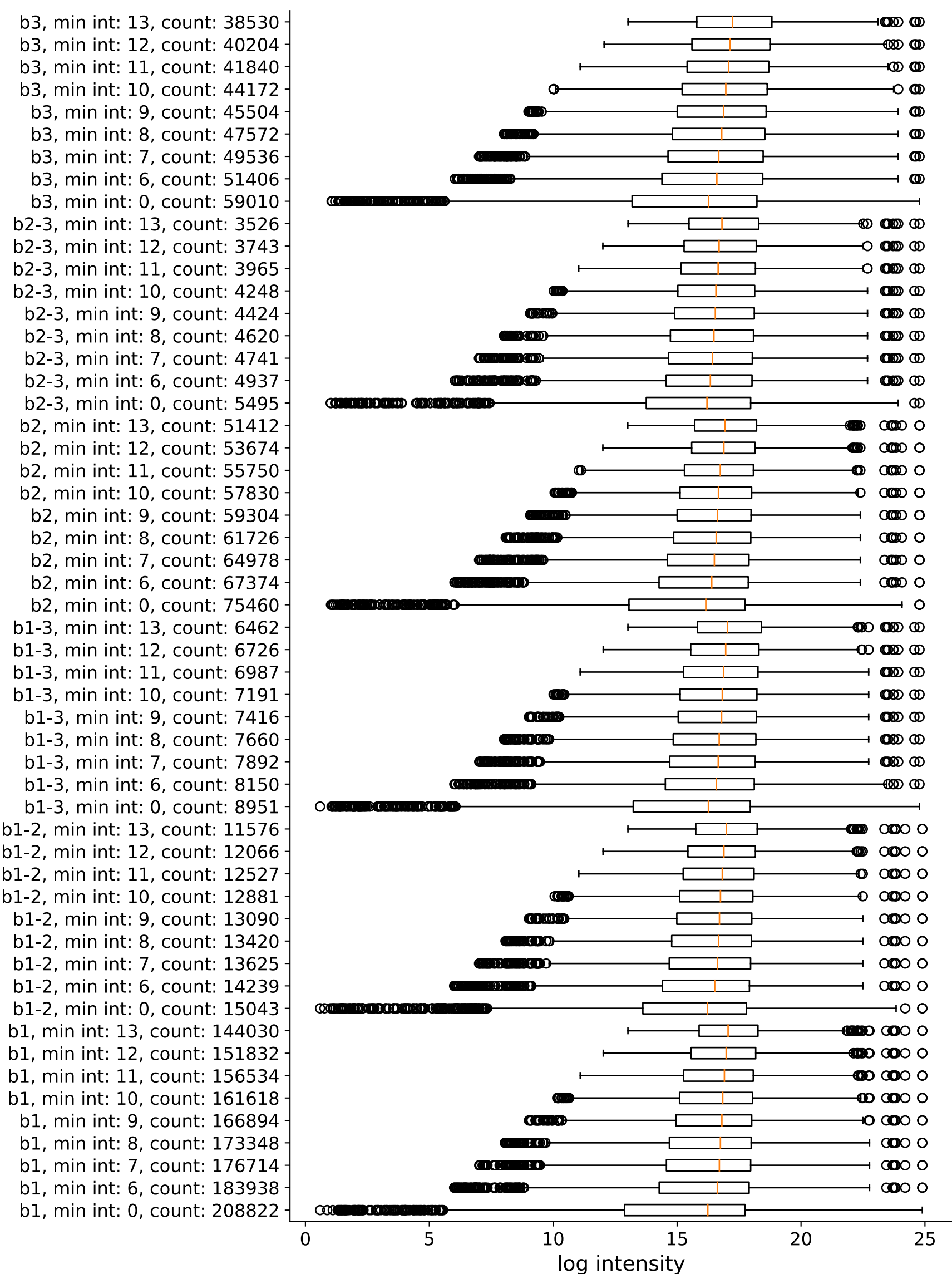

Supplement: S13 Fig — (PDF) [file pcbi.1008001.s022.pdf]

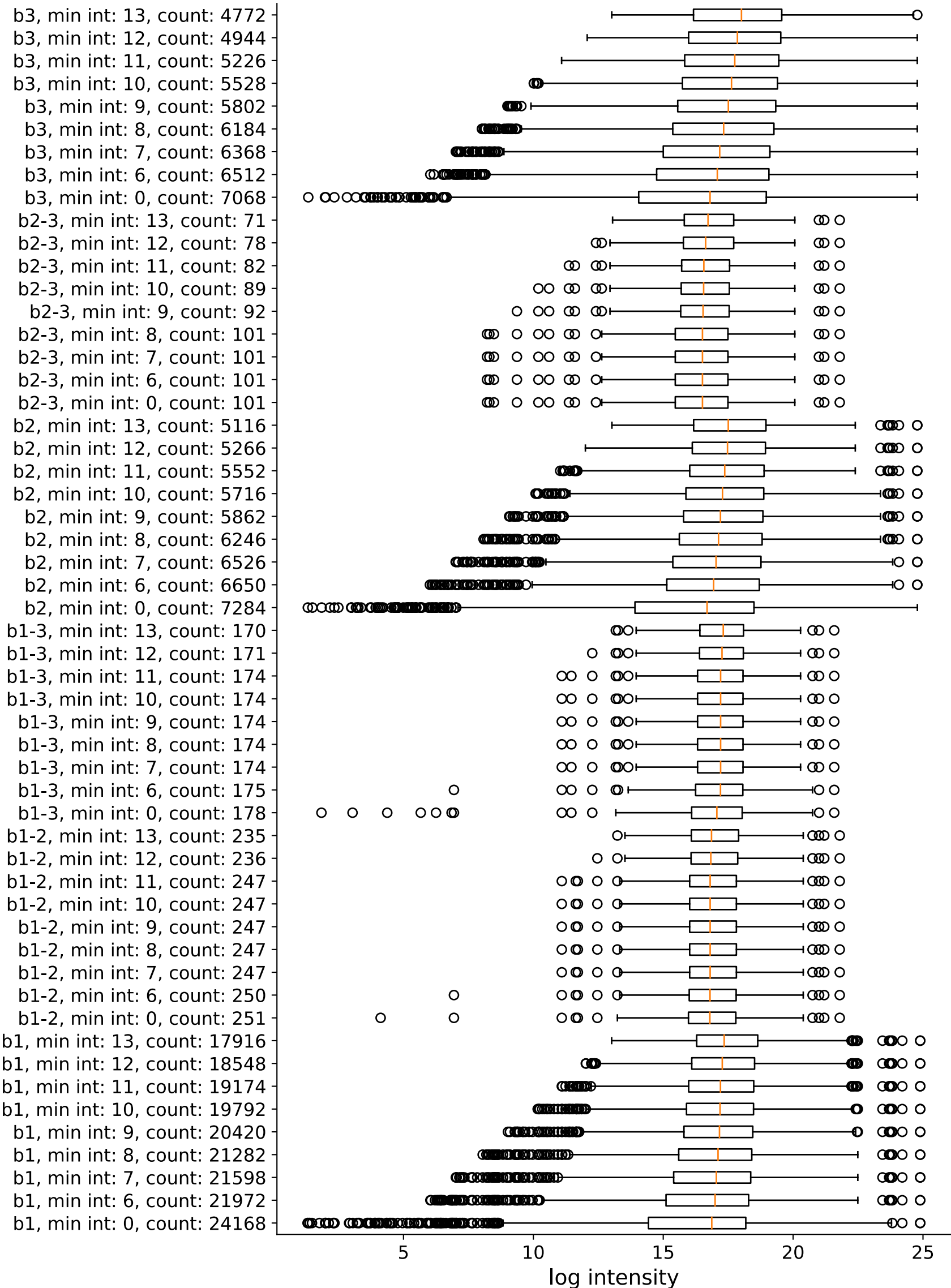

Supplement: S14 Fig — (PDF) [file pcbi.1008001.s023.pdf]

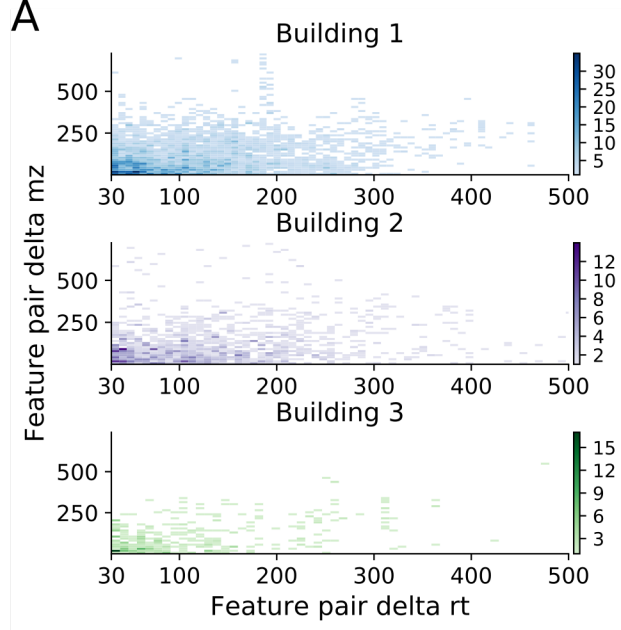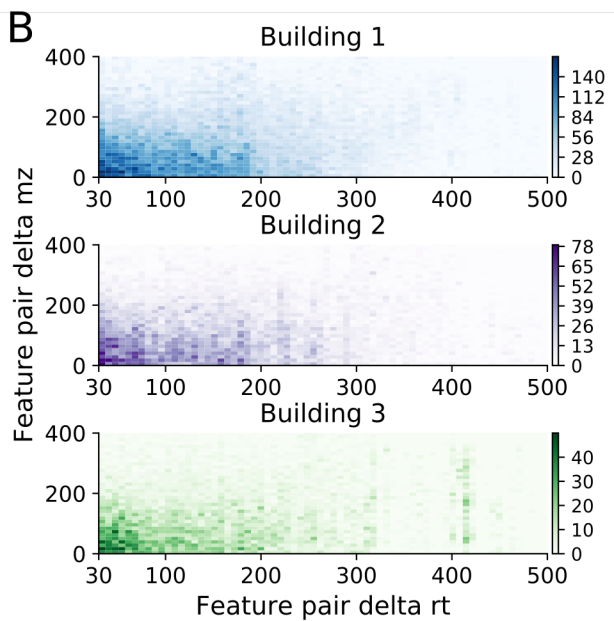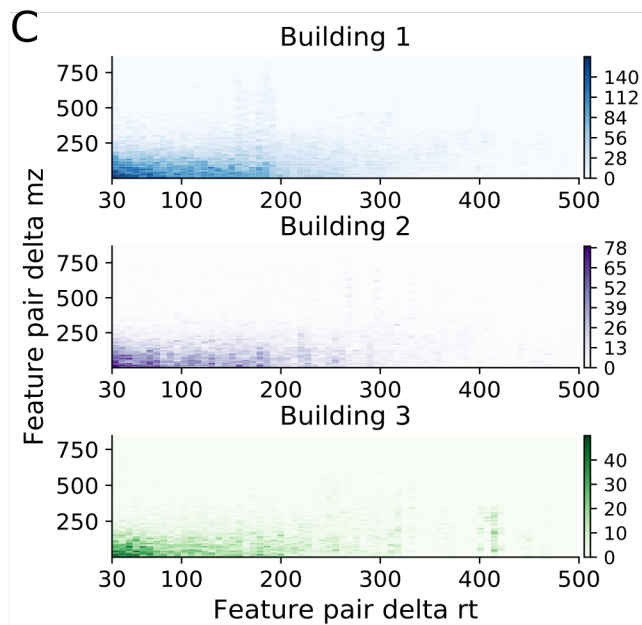

Supplement: S15 Fig — (A) Full mz and rt domain for < 1.5 distance features. (B) Reduced and (C) full domain histograms for features with similarity < 2.82 in Euclidean distance. (PDF) [file pcbi.1008001.s024.pdf]

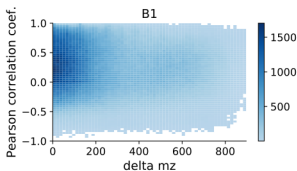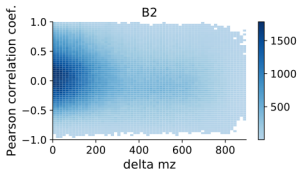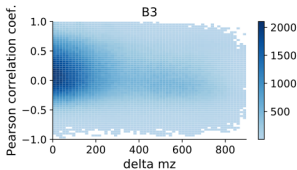

Supplement: S16 Fig — (PDF) [file pcbi.1008001.s025.pdf]

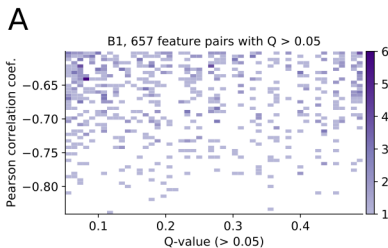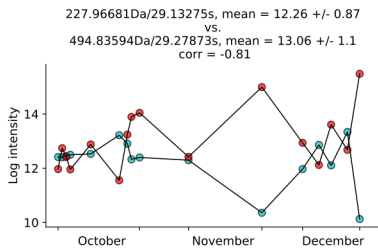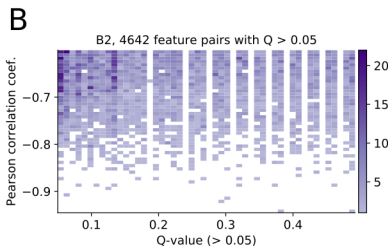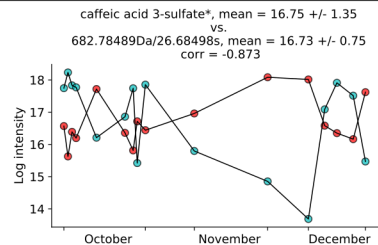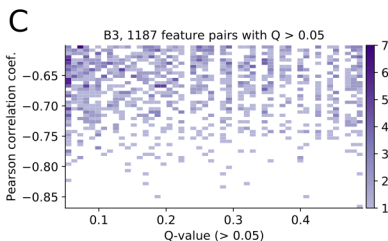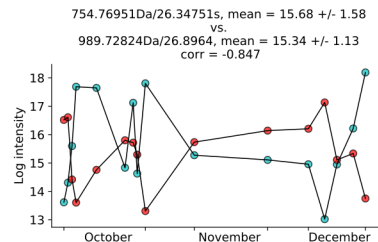

Supplement: S17 Fig — (A) Building 1 (B) Building 2, *caffeic acid 4-sulfate alternative compound. (C) Building 3 feature pairs along with example feature time course. For all time series the top label corresponds to the blue time series while the red is the second entry. ‘Mean’ refers to a feature’s through-time mean intensity with standard deviation. (PDF) [file pcbi.1008001.s026.pdf]

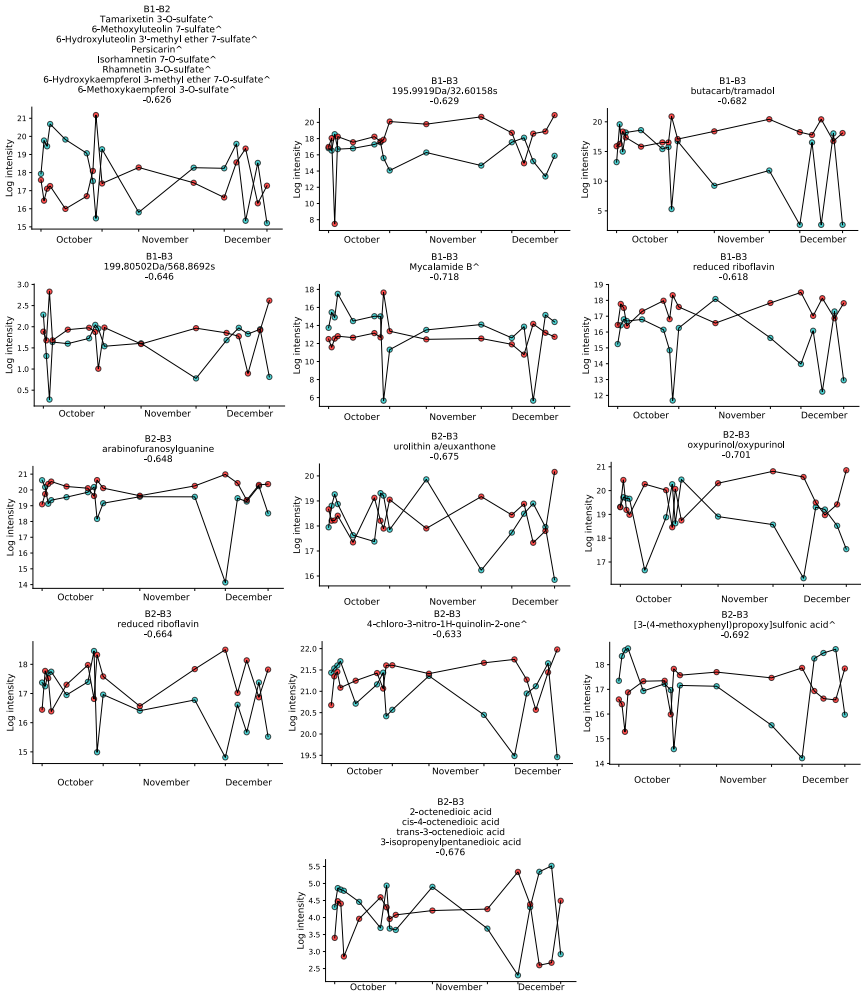

Supplement: S18 Fig — Labeled features are level 2 IDs unless marked with a ^ which corresponds to level 3. The number below the names is the feature’s building-to-building correlation value. (PDF) [file pcbi.1008001.s027.pdf]

RF ROC analysis - Average AUC 0.906+/-0.065

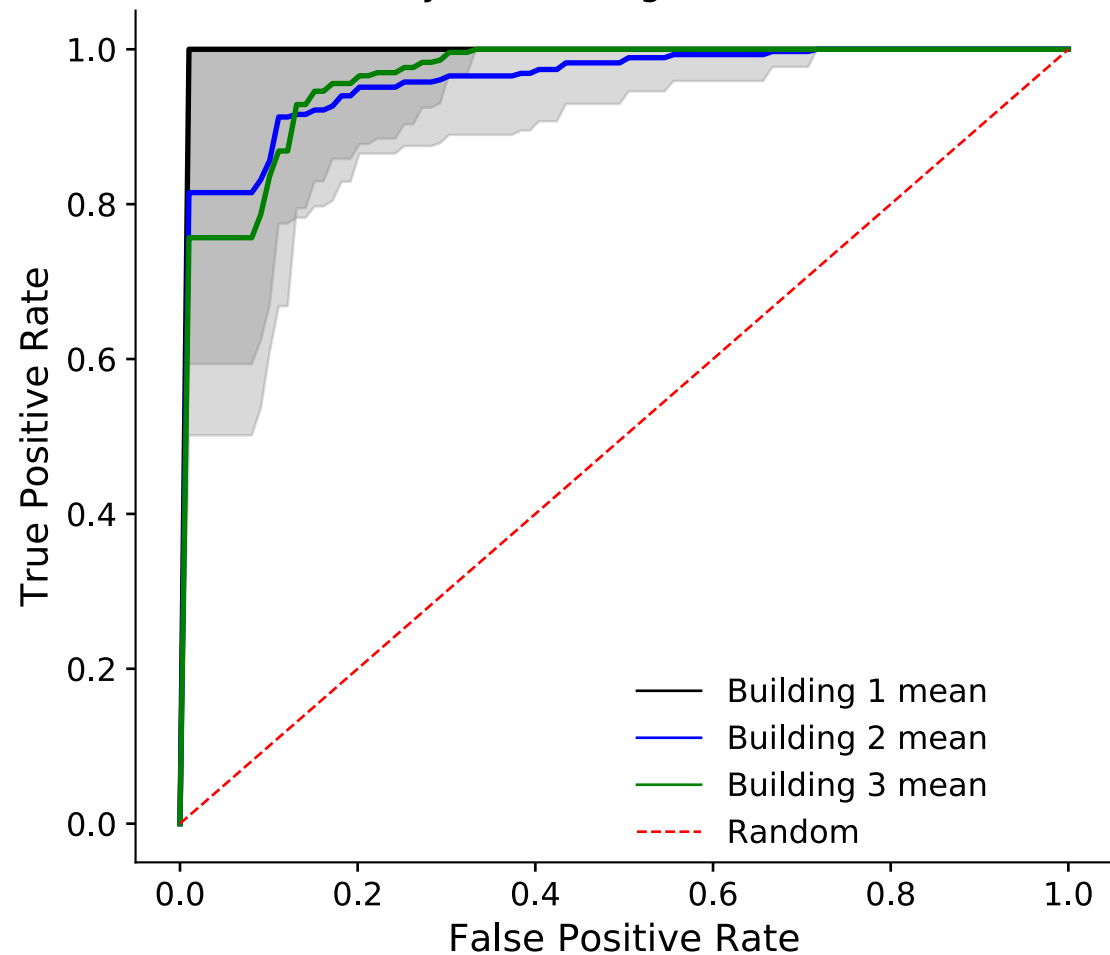

Supplement: S19 Fig — Mean of the three AUCs presented as the overall AUC. (PDF) [file pcbi.1008001.s028.pdf]

B1 - B2

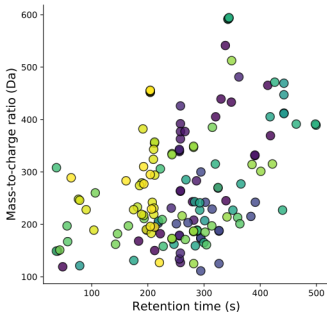

B1 - B3

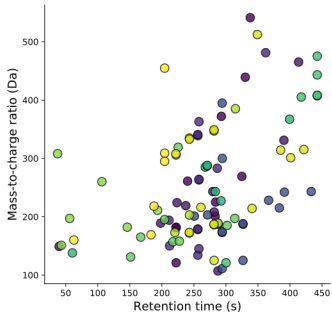

B2 - B3

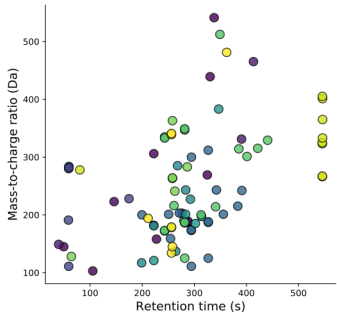

Supplement: S21 Fig — Each color corresponds to co-clustered features between the compared buildings. Co-clustering does not indicate shared dynamics between buildings, only that the features had similar dynamics internal to each building. (PDF) [file pcbi.1008001.s030.pdf]

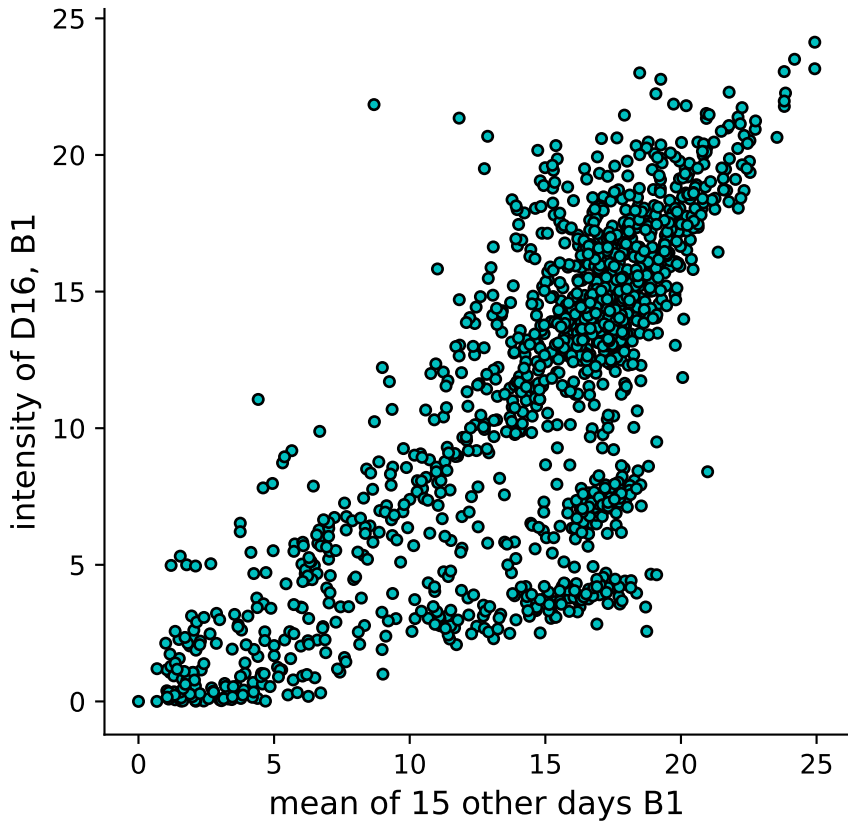

Supplement: S23 Fig — (PDF) [file pcbi.1008001.s032.pdf]

B1

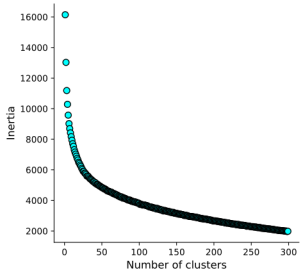

B2

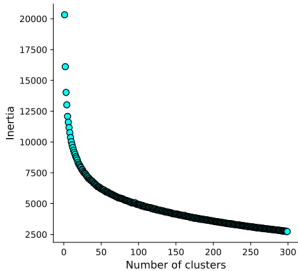

B3

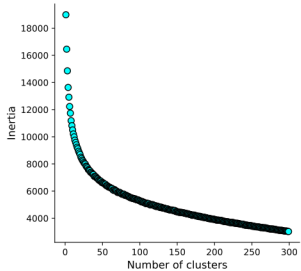

Supplement: S24 Fig — (PDF) [file pcbi.1008001.s033.pdf]

Fraction of features used

- Building 1
- Building 2
- Building 3

1.0  
0.8  
0.6  
0.4  
0.2  
0.0

0

20

40

60

80

100

Number of clusters

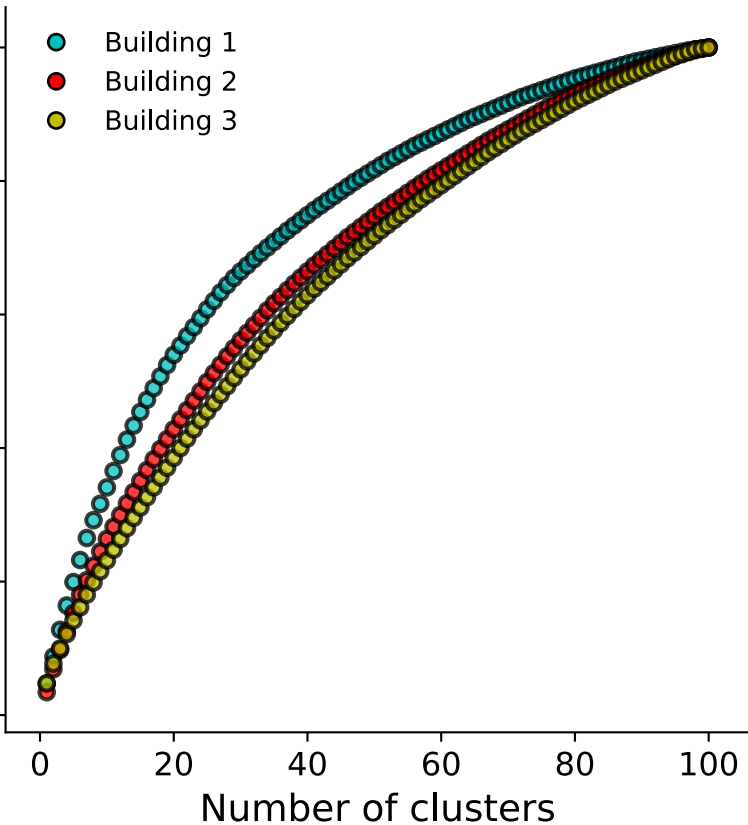

Supplement: S25 Fig — The clusters were first sorted by size and then for each number of clusters, the number of cluster members was summed for that value of combined clusters. (PDF) [file pcbi.1008001.s034.pdf]

**A** All possible rt delta (>30s)

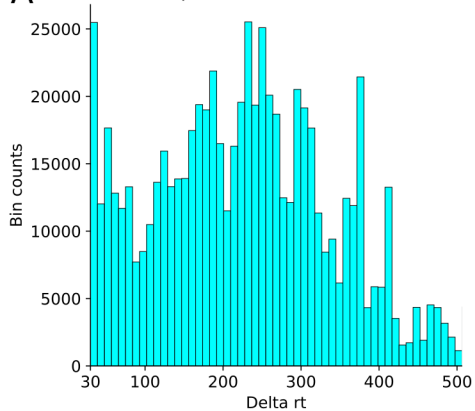

**B** All mz-rt differences

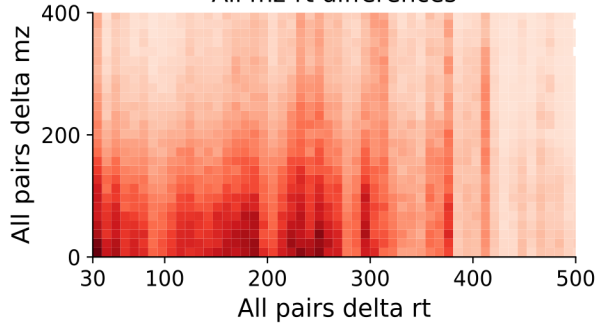

Supplement: S26 Fig — (A) All feature pairs with differences in rt of > 30s showing select delta rt bins possessing increased counts. (B) Same as in A but with a second axis of the mz differences depicting select columns with higher counts than neighboring columns. (PDF) [file pcbi.1008001.s035.pdf]
