# Supplementary material for: Longitudinal wastewater sampling in buildings reveals temporal dynamics of metabolites
Source: PLoS Comput Biol. 2020 Jun 29;16(6):e1008001. doi: 10.1371/journal.pcbi.1008001 (PMC7351223; doi:10.1371/journal.pcbi.1008001)
Supplement: S4 Fig — The following are the 38 lowest log Q-value features from ‘all features’ (top left plot): 1) 2-hydroxyethanesulfonate, 1.0; 2) (2-hydroxyethoxy)sulfonic acid**, 0.482, M-H_hmdb, C2H6O5S1; 3) l-2,3-dihydrodipicolinate, 1.0, 2-furoylglycine, 1.0; 4) n-acetyl-5-aminosalicylic acid, 1.0, 6-hydroxy-3-succinoylpyridine, 1.0; 5) pantothenol, 1.0; 6) threoninyl-proline, 1.0, gamma-glutamyl-gamma-aminobutyraldehyde, 1.0; 7) l-iditol, 1.0, d-altritol, 1.0; 8) 219.04563Da/33.92086s; 9) n-decanoylglycine, 1.0; 10) 237.93104Da/209.2619s; 11) girgensonine, 1.0; 12) (4R)-4,8-dimethylnonyl hydrogen sulfate**, 0.690, M-H_chebi, C11H24O4S1; 13) n-lauroylglycine, 1.0; 14) 4’-dihydroabscisic acid, 0.8915065391457075; 15) 267.14393Da/545.57034; 16) ferulic acid 4-sulfate, 1.0, isoferulic acid 3-sulfate, 1.0; 17) felbamate, 1.0; 18) 1,4,5-trihydroxy-Δ2,3-protoilludene**, 4.840, M-2H_Na_meta, C15H24O3, illudol**, 4.840, M-2H_Na_meta, C15H24O3, isotrichodiol**, 4.840, M-2H_Na_meta, C15H24O3, 3-hydroxylubimin**, 4.840, M-2H_Na_meta, C15H24O3, 1-Hydroxyepiacorone**, 4.840, M-2H_Na_hmdb, C15H24O3, Acorusdiol**, 4.840, M-2H_Na_hmdb’, C15H24O3, 3(4->5)-Abeo-4,11:4,12-diepoxy-3-eudesmanol**, 4.840, M-2H_Na_hmdb, C15H24O3, (4R,5S,7R,11R)-11,12-Dihydroxy-1(10)-spirovetiven-2-one**, 4.840, M-2H_Na_hmdb, C15H24O3, Apotrichodiol**, 4.840, M-2H_Na_hmdb, C15H24O3, 6alpha-Carissanol**, 4.840, M-2H_Na_hmdb, C15H24O3, alpha-Carissanol**, 4.840, M-2H_Na_hmdb, C15H24O3, Epioxylubimin**, 4.840, M-2H_Na_hmdb, C15H24O3, Dihydromyoporone**, 4.840, M-2H_Na_hmdb, C15H24O3, Piperalol**, 4.840, M-2H_Na_hmdb, C15H24O3, Zedoarondiol**, 4.840, M-2H_Na_hmdb, C15H24O3, Hydroxypelenolide**, 4.840’, M-2H_Na_hmdb’, C15H24O3, Toxin FS2**, 4.840, M-2H_Na_hmdb, C15H24O3, Urodiolenone**, 4.840, M-2H_Na_hmdb, C15H24O3, Bisacurone B**, 4.840, M-2H_Na_hmdb, C15H24O3, 2,3-Dihydroabscisic alcohol**, 4.840, M-2H_Na_hmdb, C15H24O3, 3-Methyl-5-pentyl-2-furanpentanoic acid**, 4.840, M-2H_Na_hmdb, C15H24O3, 3-Methyl-5-propyl-2- [file pcbi.1008001.s013.pdf]

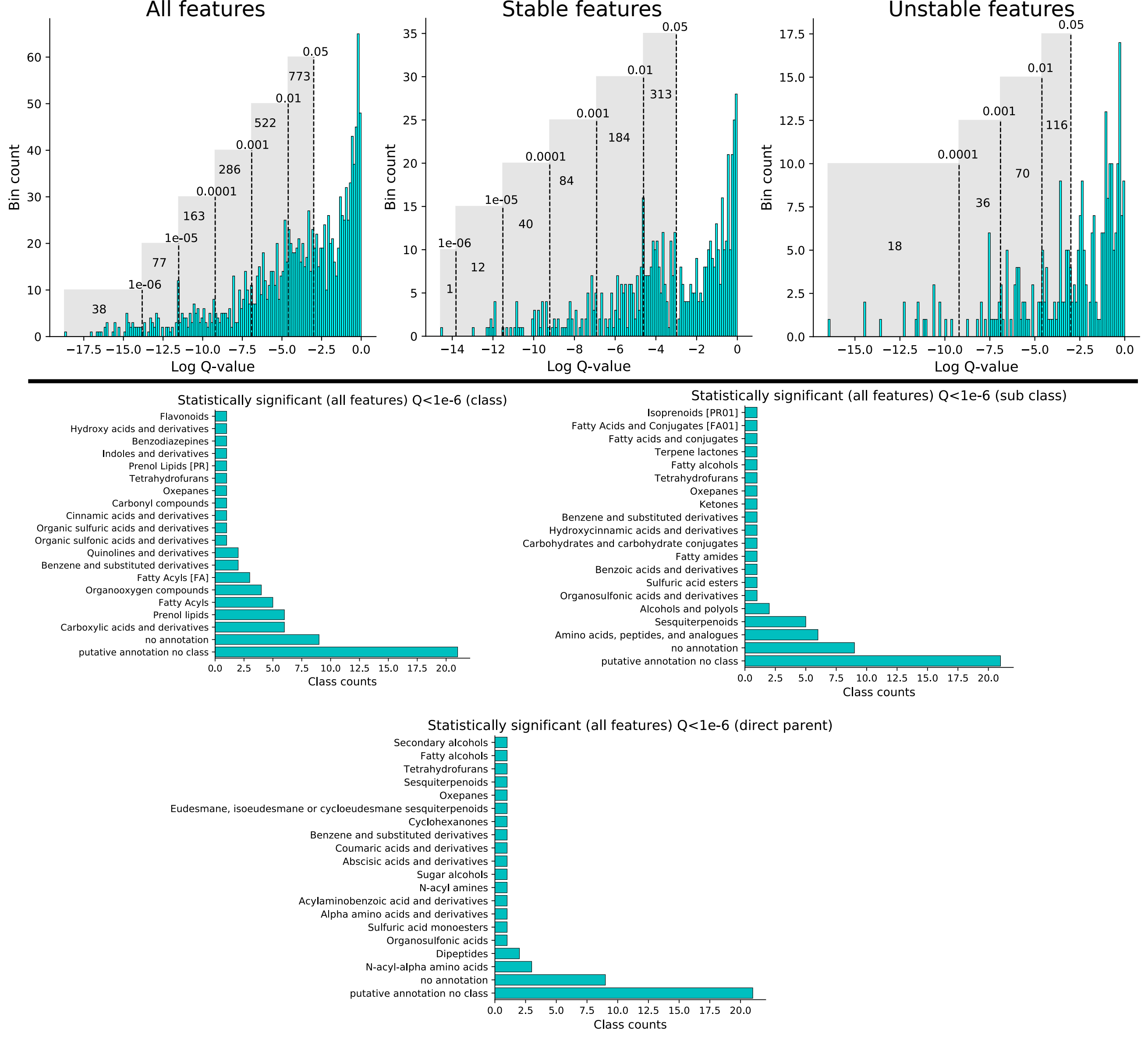
All features
Stable features
Unstable features
Bin count
Log Q-value
38
77
163
286
522
773
1e-06
1e-05
0.0001
0.001
0.01
0.05
1
12
40
84
184
313
18
36
70
116
Statistically significant (all features) Q<1e-6 (class)
Flavonoids
Hydroxy acids and derivatives
Benzodiazepines
Indoles and derivatives
Prenol Lipids [PR]
Tetrahydrofurans
Oxepanes
Carbonyl compounds
Cinnamic acids and derivatives
Organic sulfuric acids and derivatives
Organic sulfonic acids and derivatives
Quinolines and derivatives
Benzene and substituted derivatives
Fatty Acyls [FA]
Organooxygen compounds
Fatty Acyls
Prenol lipids
Carboxylic acids and derivatives
no annotation
putative annotation no class
Class counts
Statistically significant (all features) Q<1e-6 (sub class)
Isoprenoids [PR01]
Fatty Acids and Conjugates [FA01]
Fatty acids and conjugates
Terpene lactones
Fatty alcohols
Tetrahydrofurans
Oxepanes
Ketones
Benzene and substituted derivatives
Hydroxycinnamic acids and derivatives
Carbohydrates and carbohydrate conjugates
Fatty amides
Benzoic acids and derivatives
Sulfuric acid esters
Organosulfonic acids and derivatives
Alcohols and polyols
Sesquiterpenoids
Amino acids, peptides, and analogues
no annotation
putative annotation no class
Class counts
Statistically significant (all features) Q<1e-6 (direct parent)
Secondary alcohols
Fatty alcohols
Tetrahydrofurans
Sesquiterpenoids
Oxepanes
Eudesmane, isoeudesmane or cycloeudesmane sesquiterpenoids
Cyclohexanones
Benzene and substituted derivatives
Coumaric acids and derivatives
Abscisic acids and derivatives
Sugar alcohols
N-acyl amines
Acylaminobenzoic acid and derivatives
Alpha amino acids and derivatives
Sulfuric acid monoesters
Organosulfonic acids
Dipeptides
N-acyl-alpha amino acids
no annotation
putative annotation no class
Class counts
