# Supplementary material for: Longitudinal wastewater sampling in buildings reveals temporal dynamics of metabolites
Source: PLoS Comput Biol. 2020 Jun 29;16(6):e1008001. doi: 10.1371/journal.pcbi.1008001 (PMC7351223; doi:10.1371/journal.pcbi.1008001)
Supplement: S10 Fig — Compounds with ‘glucosid’ in their name, whether matched at level 2 or 3 were included in the plot. S4 Table for complete details.*hinokitiol glucoside / (3r,4r)-4,8-dihydroxy-3-((r)-2-hydroxypentyl)-6,7-dimethoxyisochroman-1-one, **methyl (3x,10r)-dihydroxy-11-dodecene-6,8-diynoate 10-glucoside / methyl 3,4-dihydroxy-5-prenylbenzoate 3-glucoside, ***5,6,7-trimethoxy-3-(3,4,5-trimethoxyphenyl)-1-benzopyran-4-one / (3’r,4’r)-3’-epoxyangeloyloxy-4’-acetoxy-3’,4’-dihydroseselin / 5-[(5-methoxycarbonyl-2-methyl-3-furanyl)methoxy]-2-methyl-3-benzofurancarboxylic acid 2-methoxyethylester/nobiletin/graminone b / hexamethylquercetagetin / 2-(2,5-dimethoxyphenyl)-5,6,7,8-tetramethoxy-4h-1-benzopyran-4-one / 7-hydroxyflavanone 7-o-beta-d-glucoside / 2-(3,5-dimethoxyphenyl)-5,6,7,8-tetramethoxy-4h-1-benzopyran-4-one, ****quercetin 3-o-alpha-l-[6‴-p-coumaroyl-beta-d-glucopyranosyl-(1->2)-rhamnopyranoside]-7-o-beta-d-glucopyranoside / kaempferol 3-o-[6-(4-coumaroyl)-beta-d-glucosyl-(1->2)-beta-d-glucosyl-(1->2)-beta-d-glucoside]. (PDF) [file pcbi.1008001.s019.pdf]

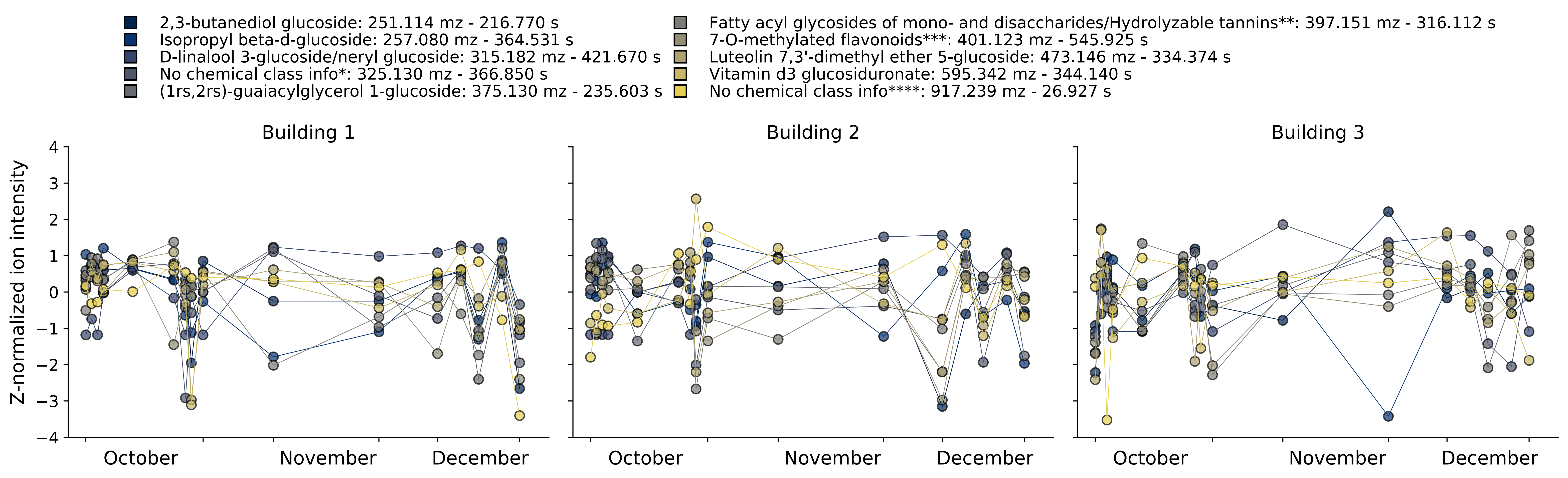
2,3-butanediol glucoside: 251.114 mz - 216.770 s
Isopropyl beta-d-glucoside: 257.080 mz - 364.531 s
D-linalool 3-glucoside/neryl glucoside: 315.182 mz - 421.670 s
No chemical class info*: 325.130 mz - 366.850 s
(1rs,2rs)-guaiacylglycerol 1-glucoside: 375.130 mz - 235.603 s
Fatty acyl glycosides of mono- and disaccharides/Hydrolyzable tannins**: 397.151 mz - 316.112 s
7-O-methylated flavonoids***: 401.123 mz - 545.925 s
Luteolin 7,3'-dimethyl ether 5-glucoside: 473.146 mz - 334.374 s
Vitamin d3 glucosiduronate: 595.342 mz - 344.140 s
No chemical class info****: 917.239 mz - 26.927 s
Building 1
Building 2
Building 3
Z-normalized ion intensity
4
3
2
1
0
−1
−2
−3
−4
October
November
December
