# Supplementary material for: Longitudinal wastewater sampling in buildings reveals temporal dynamics of metabolites
Source: PLoS Comput Biol. 2020 Jun 29;16(6):e1008001. doi: 10.1371/journal.pcbi.1008001 (PMC7351223; doi:10.1371/journal.pcbi.1008001)
Supplement: S11 Fig — (A and B) Building 1 plots. (C and D) Building 2 plots. (E and F) Building 3 plots. For all plots, vertical black lines are drawn at select distance values as indicated. (PDF) [file pcbi.1008001.s020.pdf]

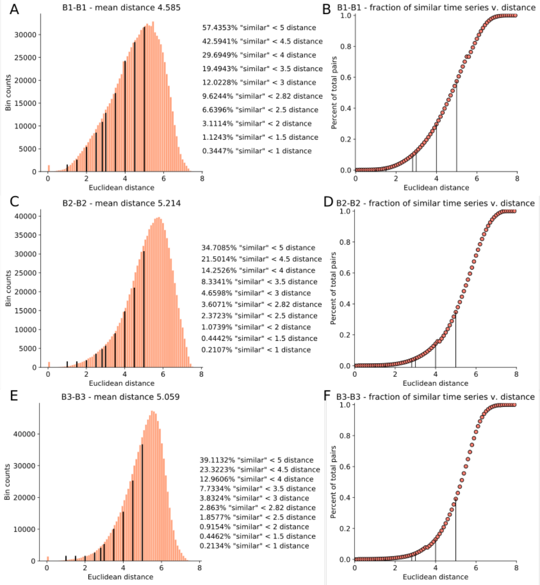
A
B1-B1 - mean distance 4.585
57.4353% "similar" < 5 distance
42.5941% "similar" < 4.5 distance
29.6949% "similar" < 4 distance
19.4943% "similar" < 3.5 distance
12.0228% "similar" < 3 distance
9.6244% "similar" < 2.82 distance
6.6396% "similar" < 2.5 distance
3.1114% "similar" < 2 distance
1.1243% "similar" < 1.5 distance
0.3447% "similar" < 1 distance
Bin counts
Euclidean distance
B
B1-B1 - fraction of similar time series v. distance
Percent of total pairs
Euclidean distance
C
B2-B2 - mean distance 5.214
34.7085% "similar" < 5 distance
21.5014% "similar" < 4.5 distance
14.2526% "similar" < 4 distance
8.3341% "similar" < 3.5 distance
4.6598% "similar" < 3 distance
3.6071% "similar" < 2.82 distance
2.3723% "similar" < 2.5 distance
1.0739% "similar" < 2 distance
0.4442% "similar" < 1.5 distance
0.2107% "similar" < 1 distance
Bin counts
Euclidean distance
D
B2-B2 - fraction of similar time series v. distance
Percent of total pairs
Euclidean distance
E
B3-B3 - mean distance 5.059
39.1132% "similar" < 5 distance
23.3223% "similar" < 4.5 distance
12.9606% "similar" < 4 distance
7.7334% "similar" < 3.5 distance
3.8324% "similar" < 3 distance
2.863% "similar" < 2.82 distance
1.8577% "similar" < 2.5 distance
0.9154% "similar" < 2 distance
0.4462% "similar" < 1.5 distance
0.2134% "similar" < 1 distance
Bin counts
Euclidean distance
F
B3-B3 - fraction of similar time series v. distance
Percent of total pairs
Euclidean distance
