# Supplementary material for: Longitudinal wastewater sampling in buildings reveals temporal dynamics of metabolites
Source: PLoS Comput Biol. 2020 Jun 29;16(6):e1008001. doi: 10.1371/journal.pcbi.1008001 (PMC7351223; doi:10.1371/journal.pcbi.1008001)
Supplement: S20 Fig — Each plot maximally shows the 20 most abundant classes. (PDF) [file pcbi.1008001.s029.pdf]

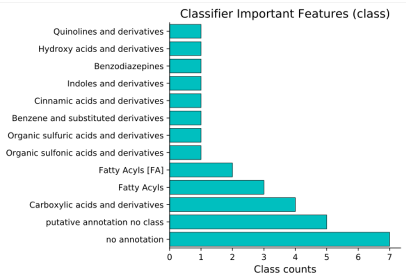
Classifier Important Features (class)
Quinolines and derivatives
Hydroxy acids and derivatives
Benzodiazepines
Indoles and derivatives
Cinnamic acids and derivatives
Benzene and substituted derivatives
Organic sulfuric acids and derivatives
Organic sulfonic acids and derivatives
Fatty Acyls [FA]
Fatty Acyls
Carboxylic acids and derivatives
putative annotation no class
no annotation
0
1
2
3
4
5
6
7
Class counts

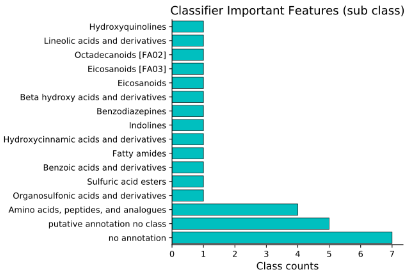
Classifier Important Features (sub class)
Hydroxyquinolines
Lineolic acids and derivatives
Octadecanoids [FA02]
Eicosanoids [FA03]
Eicosanoids
Beta hydroxy acids and derivatives
Benzodiazepines
Indolines
Hydroxycinnamic acids and derivatives
Fatty amides
Benzoic acids and derivatives
Sulfuric acid esters
Organosulfonic acids and derivatives
Amino acids, peptides, and analogues
putative annotation no class
no annotation
0
1
2
3
4
5
6
7
Class counts

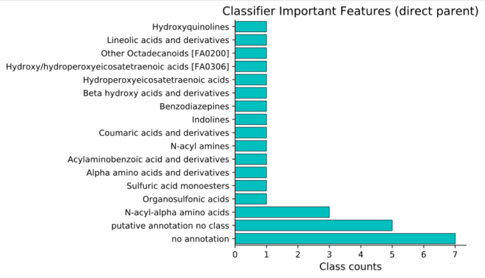
Classifier Important Features (direct parent)
Hydroxyquinolines
Lineolic acids and derivatives
Other Octadecanoids [FA0200]
Hydroxy/hydroperoxyeicosatetraenoic acids [FA0306]
Hydroperoxyeicosatetraenoic acids
Beta hydroxy acids and derivatives
Benzodiazepines
Indolines
Coumaric acids and derivatives
N-acyl amines
Acylaminobenzoic acid and derivatives
Alpha amino acids and derivatives
Sulfuric acid monoesters
Organosulfonic acids
N-acyl-alpha amino acids
putative annotation no class
no annotation
0
1
2
3
4
5
6
7
Class counts
