# Supplementary material for: Longitudinal wastewater sampling in buildings reveals temporal dynamics of metabolites
Source: PLoS Comput Biol. 2020 Jun 29;16(6):e1008001. doi: 10.1371/journal.pcbi.1008001 (PMC7351223; doi:10.1371/journal.pcbi.1008001)
Supplement: S22 Fig — Autocorrelation was calculated at all lag times for the data set Ticks show the first and last sampling dates of each month (October, November, December). Autocorrelation was calculated with statsmodels.tsa.stattools.acf. (PDF) [file pcbi.1008001.s031.pdf]

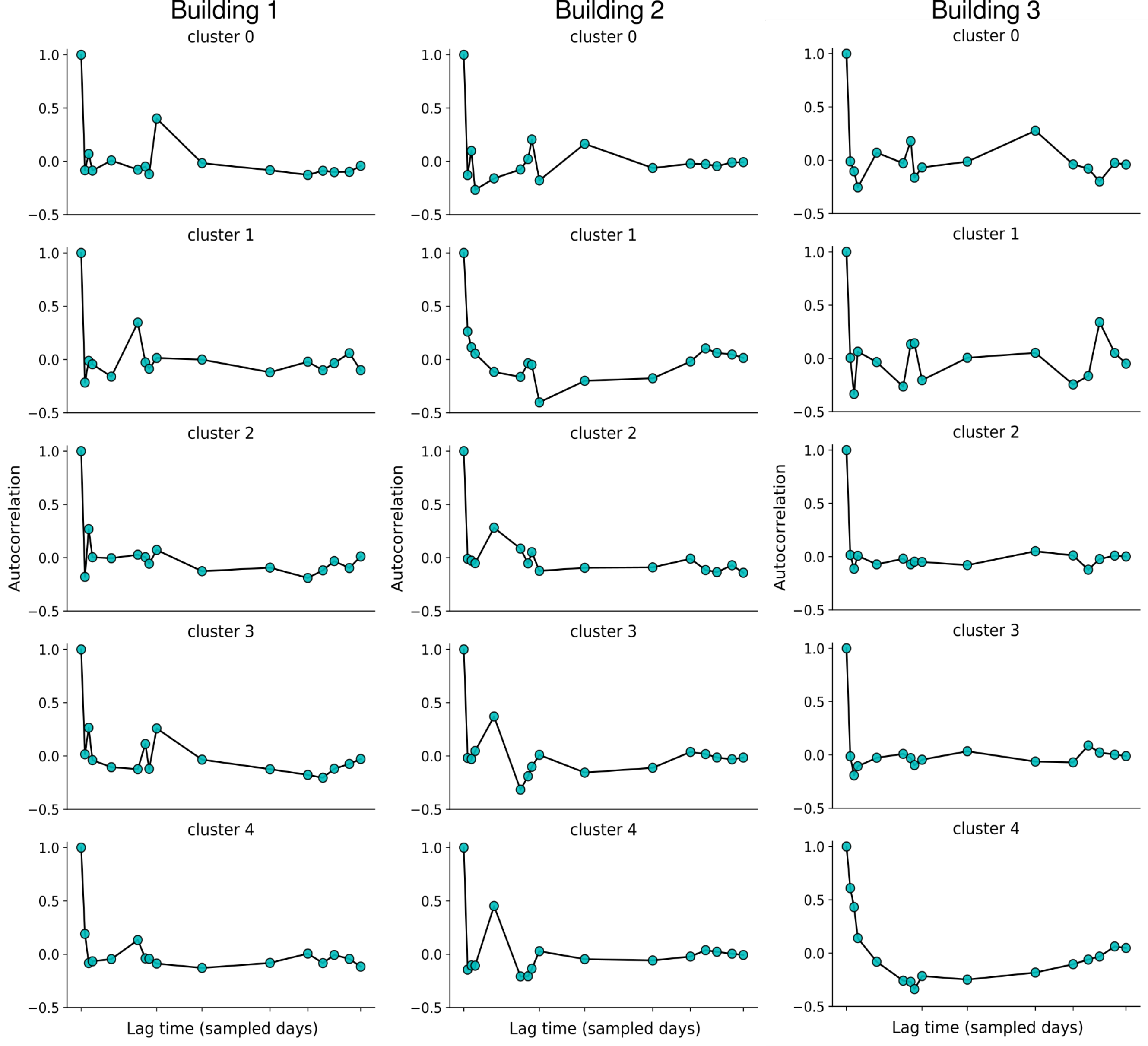
Building 1
Building 2
Building 3
cluster 0
cluster 1
cluster 2
cluster 3
cluster 4
Autocorrelation
Lag time (sampled days)
1.0
0.5
0.0
−0.5
