# Supplementary material for: Longitudinal wastewater sampling in buildings reveals temporal dynamics of metabolites
Source: PLoS Comput Biol. 2020 Jun 29;16(6):e1008001. doi: 10.1371/journal.pcbi.1008001 (PMC7351223; doi:10.1371/journal.pcbi.1008001)
Supplement: S1 Text — Detailed methods, additional text and commands for reproducing this work using associated code and data. (DOCX) [file pcbi.1008001.s036.docx]

**Additional data processing, methods, and text**

The following is an outline of how to process the data (similar information is on the associated Github pages), extract the primary feature table and associated secondary mass spectra (MS2) and then name the features from this XCMS output, for use in downstream analysis.

*Feature naming*

For naming we note that we attempted to include possible isotopes, primarily multiple C13 peaks; however, we find many of these labels to not be realistic as most of the time only the C13 version of a compound would be found and not the C12, this makes little sense chemically and thus we urge the user to not pay attention to these labels and remove them. Additionally, we have found ‘hydrate’ compounds as well as salts including ‘sodium’ or ‘potassium’ in some database names, these too were discarded. Additional reasons for a name to be discarded can be found in S1 Table.

*Feature intensity sum minimum*

The value of 100 was a heuristic set allowing for features to either be present at high levels on relatively few days (i.e., requiring a feature to appear only 5–10 times across all samples with larger intensities) or consistently found at low levels in the majority of samples. This value can and should be tuned for different data sets or analyses.

*Feature intensities for D16, B1*

Only select features show decreased intensities relative to their mean values while the majority of features show similar intensities (S23 Fig). Because the majority of features are not decreased relative to their mean values, and instead certain features appear to primarily be altered this day was included in the analysis and not removed as an outlier.

*Analysis of the choice of number of K-means clusters*

We used 100 centers, despite an elbow plot analysis that suggested ~50 centers (S24 Fig). Given that between 18 and 28% of features could not be accounted for by the 50 largest clusters when using 100 centers (S25 Fig), using only 50 centers would lead to overly inclusive clusters that may lose dynamics information. The larger 100 centers captured the most general patterns (e.g., the top 50 clusters) but also allowed for small clusters to accommodate features with different dynamics while not unnecessarily splitting clusters that a larger value would do.

*Column and instrumental origins of specific delta rt values with increased bin counts*

The increased bin counts come from the fact that many compounds (across the full mz range) elute between 26s and 32s in the liquid chromatography gradient since a C18 column was used. Thus, for any other time window where an increased number of features appear, there will be a higher number of pairs where one is from the 26-32s window. This may end up creating vertical bands in 2D histogram plots like those shown in S26 Fig and possibly Fig 5C Building 3.

*File conversion*

To convert the file to .mzML format the following command was used on a Windows operating system, this was run in the directory that contained the mscovert.exe program, otherwise the script can be modified to accept an input msconvert.exe path:

.\path\to\msconvert_ee.py .\path\to\data

*XCMS processing*

To process the .mzML data, the following command line script is a template to run the full_ipo_xcms.py script that wraps both IPO and XCMS:

python full_ipo_xcms.py --data_type 'mzML' \

--in_path='/path/to/data/' \

# if IPO out files exist, otherwise remove the following line (ie for the first time running):

--ipo_files='IPO_1.out IPO_2.out IPO_3.out' \

--data_file='name_of_file_with_path_and_names_to_all_mzMLs.txt' \

--acq_mode='negative' \

--csv_out='out_file_name.csv' \

--out_path='/path/where/you/want/the/data/' \

--log_file='out_file_log_name.log'

This generated both the .csv file with features and their intensities as well as the .mgf file for metabolite naming.

1) Use parsing_metabolite_dbs.py

This will read in and process the various databases (HMDB [[1](https://www.zotero.org/google-docs/?lCyvCw)], MetaCyc [[2](https://www.zotero.org/google-docs/?9f0z28)], ChEBI [[3](https://www.zotero.org/google-docs/?xCl5kn)] and LIPID MAPS [[4](https://www.zotero.org/google-docs/?mYf5J8)]: hmdb_metabolites.xml, compounds.dat, ChEBI_complete.sdf and structures.sdf) but you must run each individually with the appropriate command line flag after downloading the appropriate databases, these are not included in our data or github page.

2) Use mapping_mz_to_metabolites.py

This will perform the mapping from mz features to small molecule names in the databases. It makes a csv file for each dataset and outputs them to a user specified directory. Example command line (all options are detailed in the script itself and besides path, this command was used for this manuscript):

Python ./mapping_mz_to_metabolites.py -f './path/in_file_after_ipython_feat_processing.csv' -p 10 -c 1 -m 'negative' -o './path/out_file.csv'

3) Use metabolite_calling.py

This will go through the csv file from the previous step, cluster by defined retention time (rt) for peak grouping, find similar chemicals and call the best (if possible) metabolite. The metabolite calling or ‘ranking’ had the following priorities: [M-H]^-^ > [M+Cl]^-^ = [M-H-H_2_O]^-^ = [M]^-^ > [2M-H]^-^ = [M-2H+Na^+^]^-^ = [M-2H+K^+^]^-^ = [M+(1-3)^13^C-H]^-^. This creates a file with the following general name: all_voted_in_file_from_step_2.csv An example command line is:

python ./metabolite_calling.py -t 5 -r 10 -f "./path/in_file_from_step_2.csv" -s ',' -o './path/'

To get MS-MS verification:

1) run parse_mgf_comb_feat_prep_metfrag.py on the all_voted_* file from the voting above. This must be run in the same folder as the Metfrag program [[5](https://www.zotero.org/google-docs/?GMI4sf)]. It runs in parallel all the metfrag programs generated for 3 (or fewer if that’s all there are) MS2 spectra for a single mz/rt pair for each possible adduct. This requires you to have the extra database files: hmdb_2017-07-23.csv, kegg_2017-07-23.csv, lipidmaps.csv (not provided since we do not own this data). Also, **it requires a subfolder named msms_out**, this is where all the output files will be placed. Example command line:

python parse_mgf_comb_feat_prep_metfrag.py -f './all_voted_in_file_from_step_2.csv' -c 2 -r 3 -m './mgf_file_from_xcms.mgf' -v 'true'

2) run combine_metfrag_w_votes.py

This will combine the output of the metfrag program (lots of individual files with the voted results) to find the best metabolite names. Examples command line:

python combine_metfrag_w_votes.py -f './all_voted_in_file_from_step_2.csv' -c 2 -r 3 -m './path/to/msms_out/' -v 'true' -n 'True' -o './final_named_metabolites_ordered.csv'

3) To match the named metabolites to the features and their intensities use the data_processing.ipynb (along with its primary function of finding the best features to work with) and follow all the way to the end and save the final output file ‘partly_cleaned_metals_combined_mz_rt_metfrag_votes_isotopes_mzrt_named.csv’. This removes most implausible compounds according to Table S1.

To perform data analysis and reproduce results of the papers follow the four following notebooks with save = True and processed = False for the first time through and then reversed so as not to retrain the various models. Note, results may differ due to different random seed for algorithms. Use the following workflow:

1) data_processing.ipynb

2) [splitting_stable_unstable_metabs_pub.ipynb](http://localhost:8888/notebooks/underworlds_TC/analysis/splitting_stable_unstable_metabs_pub.ipynb)

3) [Building_and_day_classification.ipynb](http://localhost:8888/notebooks/underworlds_TC/analysis/Building_and_day_classification.ipynb)

4) [temporal_dynamics_analysis.ipynb](http://localhost:8888/notebooks/underworlds_TC/analysis/temporal_dynamics_analysis_2.ipynb)

*Theoretical waste stream mixing suggests dynamics are lost with a small number of additional sources.*

Simulating wastestream combination or longer sampling periods suggested that the unique dynamics observed in single-building, 3-hour samples are lost with the addition of only a few waste streams. We combined the data from the three buildings with a desired number of modeled waste streams, clustered the combination and fit each cluster with a Gaussian Process (GP). Repeating this protocol with different numbers of combined waste streams suggested that major cluster dynamics are dampened when only tens of waste streams are combined, and lost with more than 50 (S9 Fig.). GP modeling of the combined data suggested that the GP uncertainty rapidly drops as additional waste streams are mixed. Under our modeling assumptions, features will be found at their expected values with high probability and minimal day-to-day variation (S9 Fig A-E). To measure how the differences between clusters decrease with additional waste streams, we calculated the summed all-to-all cluster mean Euclidean distance. This demonstrated that cluster differences are effectively lost when waste from 50–100 sources is merged (S9 Fig F). Molecules that are rarely observed or that come from a small number of sources may still display significant dynamics; however, the majority of features will generally be observed at statistical averages. This underscores the importance of short, upstream collection to obtain relevant public health information.

*Waste stream simulations and mixing methods*

To match experimental data as accurately as possible, for each waste stream simulation we sampled 100 cluster means for the 16 shared days using a Cauchy distribution (scipy.stats.cauchy, loc=0, scale=0.3, size=1 and resampled if a value greater than 3.5 was obtained). We then sampled the number of intensity spikes (positive and negative) for the new wastestream from a Gaussian distribution (random.gauss, mean=0, standard deviation=1) and took the absolute value of the integer representation. We then randomly determined which days would have the spikes and the number of clusters across which this spike would occur (they normally occured over many clusters in one day) using 20% of the number of clusters (20) times the absolute, integer value of a Gaussian of 0 mean and unit standard deviation. The exact clusters with the spiking dynamics were probabilistically determined for each day independently using a decreasing exponential probability (0.002+e^-i^, where i is the cluster rank), the clusters were looped over (from largest to smallest) with a value in [0.0, 1) randomly chosen, if the value was less cluster’s probability threshold it was included in the spiking cluster. This was repeated until the total number of spiking clusters was satisfied. For these linked clusters, the mean values were resampled from the original cauchy distribution but forced to be between 1.75 and 3.

Many of the non-spiking feature intensities were similarly conserved across clusters. For this we sampled the number of days with ‘correlated’ features using the absolute integer value of a Gaussian with 0 mean and 3 for its standard deviation. The number of correlated clusters was performed as before but with a multiplicative factor of 70%; days of correlation were then probabilistically chosen according with 0.002+0.8e^-0.8i^ representing a cluster’s probability being included (this spreads the probability out over more clusters, especially to the smaller ones). For these clusters, new means were chosen from the original cauchy distribution but forced into the range of being less than 1.75.

Finally, once all cluster means were sampled, 1425 features were drawn (with cluster sizes proportional to the average cluster size in the three real buildings) using a Gaussian distribution with the cluster mean for each cluster and a scale of 0.3. All values were forced to be less than 3.2 as intensities of this magnitude were rarely if ever observed in the three real data sets.

For mixing, this waste stream profile generation process was repeated a select number of times. For all numbers of mixed waste streams, the three real buildings were mixed at random percentages with the simulated data to create a single mixed data set which was then clustered with K-means (100 clusters), and each cluster fit with a GP. The calculation of the all cluster mean to all cluster mean distance was performed using the distance_matrix function and then summed. To get statistical parameters for this process, the analysis was repeated 10 times to calculate a mean and standard deviation for each number of waste streams mixed.

1. **References**

[1. Wishart DS, Jewison T, Guo AC, Wilson M, Knox C, Liu Y, et al. HMDB 3.0—The Human Metabolome Database in 2013. Nucleic Acids Res. 2013 Jan 1;41(D1):D801–7.](https://www.zotero.org/google-docs/?ItlVZS)

[2. Caspi R, Billington R, Fulcher CA, Keseler IM, Kothari A, Krummenacker M, et al. The MetaCyc database of metabolic pathways and enzymes. Nucleic Acids Res. 2018 Jan 4;46(D1):D633–9.](https://www.zotero.org/google-docs/?ItlVZS)

[3. Hastings J, Owen G, Dekker A, Ennis M, Kale N, Muthukrishnan V, et al. ChEBI in 2016: Improved services and an expanding collection of metabolites. Nucleic Acids Res. 2016 Jan;44(D1):D1214-9.](https://www.zotero.org/google-docs/?ItlVZS)

[4. Sud M, Fahy E, Cotter D, Brown A, Dennis EA, Glass CK, et al. LMSD: LIPID MAPS structure database. Nucleic Acids Res. 2007 Jan;35(Database issue):D527-532.](https://www.zotero.org/google-docs/?ItlVZS)

[5. Ruttkies C, Schymanski EL, Wolf S, Hollender J, Neumann S. MetFrag relaunched: incorporating strategies beyond in silico fragmentation. J Cheminformatics. 2016 Jan 29;8(1):3.](https://www.zotero.org/google-docs/?ItlVZS)
